# Economic Evaluation of Direct Oral Anticoagulants Versus Low-Molecular Weight Heparin for Cancer-Associated Thrombosis in a Thai University-Affiliated Hospital

**DOI:** 10.3390/jcm15010212

**Published:** 2025-12-27

**Authors:** Thanyarat Chaiwattanakowit, Nutnicha Pinitpracharome, Witoo Dilokthornsakul, Tananchai Akrawikrai, Piyameth Dilokthornsakul

**Affiliations:** 1 Master’s Degree Program in Pharmacy Management, Faculty of Pharmacy, Chiang Mai University, Chiang Mai 50200, Thailand; thanyarat_ch@cmu.ac.th; 2 Department of Pharmacy, Chiang Mai University Hospital, Faculty of Medicine, Chiang Mai University, Chiang Mai 50200, Thailand; nutnicha_pi@cmu.ac.th; 3 Center for Medical and Health Technology Assessment (CM-HTA), Department of Pharmaceutical Care, Faculty of Pharmacy, Chiang Mai University, Chiang Mai 50200, Thailand; witoo.dilok@cmu.ac.th; 4 Division of Hematology, Department of Medicine, Rajavithi Hospital, College of Medicine, Rangsit University, Bangkok 10400, Thailand; tananchai.ak@rsu.ac.th

**Keywords:** cost-effectiveness, cost-utility analysis, Markov model, cancer-associated thrombosis, venous thromboembolism, Thailand, direct oral anticoagulants, low-molecular-weight heparin, apixaban

## Abstract

**Background/Objectives:** Venous thromboembolism (VTE) is an important global health concern associated with considerable morbidity and mortality. Despite established guidelines for VTE treatment, there is a gap between clinical recommendations and their implementation in practice because of limited accessibility, particularly in low- and middle-income countries and among patients with cancer. This study aimed to assess the cost-effectiveness of direct oral anticoagulants (DOACs) on VTE in patients with cancer at a Thai university-affiliated hospital. **Methods:** A cost–utility analysis using a Markov model was developed to estimate costs and quality-adjusted life-years (QALYs) of DOACs and low-molecular weight heparin (LWMH) in Thai patients with cancer aged over 60 years. The model with eight health states, including CAT on treatment, pulmonary embolism (PE), deep vein thrombosis (DVT), clinically relevant nonmajor bleeding (CRNMB), non-intracranial hemorrhage major bleeding (non-ICH MB), intracranial hemorrhage (ICH), off treatment, and any death, was developed with a one-month cycle length and used to estimate costs and health outcomes from a societal perspective with a lifetime horizon. The efficacy and safety of DOACs compared to LMWH were obtained from a network meta-analysis, while the costs were based on a Thai university hospital database. All costs and outcomes were discounted at 3%, and the Thai societal willingness-to-pay (WTP) threshold (THB 160,000 per QALY gained) was applied. The incremental cost-effectiveness ratio (ICER) was calculated to compare costs and QALYs of the interventions. **Results:** The total lifetime cost of LMWH was THB 70,928 (USD 2,163), while for apixaban, dabigatran, edoxaban, and rivaroxaban, the costs were THB 26,323 (USD 803), THB 33,667 (USD 1,027), THB 29,570 (USD 902), and THB 22,310 (USD 680), respectively. The QALYs for LMWH, apixaban, dabigatran, edoxaban, and rivaroxaban were 0.771, 0.775, 0.746, 0.759, and 0.770 QALYs, respectively. Compared to LMWH, apixaban provided 0.004 additional QALYs, with a decreased cost of THB 44,605 (USD 1,360), resulting in reduced expenses. On the other hand, dabigatran, edoxaban, and rivaroxaban were also associated with lower lifetime costs but reduced life-years and QALYs when compared with LMWH. **Conclusions:** This study revealed that apixaban is likely to be the preferred option for treating patients with CAT. However, policy decision-making process should take into account the uncertainties related to the implementation of this practice.

## 1. Introduction

Thrombosis, encompassing both arterial and venous events, is a major global health burden [1] and remains an important cause of death, accounting for approximately one in four deaths worldwide. Venous thromboembolism (VTE), including deep vein thrombosis (DVT) and pulmonary embolism (PE), is a major clinical problem. The incidence of cancer-associated thrombosis (CAT) has notably increased over time, rising from 1.0 percent in 1997 to 3.4 percent in 2017 [2]. Patients with cancer have a reported nine-fold increased risk of VTE compared to the general population [3]. VTE is the second leading cause of death among patients with cancer, contributing to increased morbidity, mortality, and disability [4].

Anticoagulant therapy is crucial for reducing recurrent VTE (rVTE), enhancing quality of life, and improving survival rates in cancer patients [5]. Anticoagulant therapies used for VTE treatment include low-molecular weight heparin (LMWH) such as enoxaparin, vitamin K antagonists (VKAs) such as warfarin, and direct oral anticoagulants (DOACs) including apixaban, dabigatran, edoxaban, and rivaroxaban. DOACs have been developed to overcome the limitation of warfarin which needs close laboratory monitoring based on the international normalized ratio (INR) to ensure therapeutic efficacy and safety. In addition, DOACs could offer fixed-dose administration, which provides more convenience to both patients and healthcare providers when administering the medications. Moreover, DOACs also have an advantage over LMWH by offering oral administration, while LMWH needs the subcutaneous injection which could limit the patient’s acceptability to the medication due to its inconvenience and adherence to the medication [6]. However, the selection of appropriate therapy for VTE should always prioritize patient suitability and comorbidities. Currently, DOACs have not been listed in the Thailand’s National List of Essential Medicines (NLEM) due to their prices. Despite increasing clinical evidence supporting the use of DOACs for CAT, no cost-effectiveness evaluations have been conducted in Thailand. This lack of local economic evidence represents a critical gap for policy decision-making process, resulting in a limited access to DOACs.

Previous economic evaluations of other countries have shown that DOACs are more cost-effective than LWMH for patients with CAT. A cost-effectiveness analysis study in the Netherlands [7] found that rivaroxaban was a cost-effective alternative to dalteparin in patients with CAT. Similarly, an economic comparison of edoxaban and LMWHs in Brazil [8] found that edoxaban was a cost-effective and cost-saving alternative to dalteparin for treating CAT. In the United States [9,10], rivaroxaban or edoxaban use for six months among patients with CAT reported that DOACs reduced overall costs but did not improved QALY compared with dalteparin. Additionally, a study from China [11] also reported that DOACs were a cost-effective treatment for CAT. However, the cost-effectiveness results from other countries could not be directly transferred to Thailand due to the differences in healthcare systems, drug prices, clinical practices, hospitalization, diagnostic tests, physician fees, and patient characteristics. In addition, there is no cost-effectiveness study of any DOAC for patients with CAT in Thailand, resulting in the uncertainty of the value of DOACs for patients with CAT in the Thai context. Therefore, this study aimed to assess the cost-effectiveness of four DOACs compared with LMWH for the treatment of CAT in a Thai university-affiliated hospital of cancer patients aged over 60 years from a societal perspective.

## 2. Methods

### 2.1. Overall Description and Study Population

A cost–utility analysis using a state-transition Markov model was developed to simulate disease progression in Thai patients with CAT. The starting age of 60 years was selected based on the average age of Thai patients with CAT [12]. No specific cancer types or stages were modeled separately; instead, the model represented an average Thai patient with CAT, and was consistent with available epidemiological data.

### 2.2. Interventions and Comparator

Four DOACs are available in Thailand. Regimen dosages were selected according to standard recommendations for the treatment of CAT based on clinical trials; international guidelines including apixaban, rivaroxaban, edoxaban, and dabigatran were individually compared with enoxaparin as the LMWH comparator. Enoxaparin was selected because it is the most commonly used LMWH in Thai clinical practice and widely available in public hospitals. Although dalteparin is commonly used in landmark CAT trials, it is less accessible and not routinely prescribed in Thailand. Warfarin was excluded because current guidelines no longer recommend it as first-line therapy for CAT.

DOACs and LMWH are preferred due to their superior efficacy and fewer drug–food and drug–drug interactions compared with warfarin, making warfarin a secondary option when DOACs or LMWH are not suitable. The cost–utility of each DOAC and enoxaparin as a comparator were assessed. The regimen used for VTE treatment for each DOAC were as follows [13,14,15,16]. Enoxaparin: 1 mg/kg subcutaneously every 12 h for 6 months. Dabigatran: Initial treatment with subcutaneous heparin/LMWH for 5 days, followed by dabigatran 150 mg orally twice daily for 6 months. Rivaroxaban: Initial treatment with rivaroxaban 15 mg orally twice daily for 21 days, then 20 mg orally once daily for 6 months. Apixaban: Initial treatment with apixaban 10 mg orally twice daily for 7 days, then 10 mg orally once daily for 6 months. Edoxaban: Initial treatment with subcutaneous heparin/LMWH for 5–10 days, followed by edoxaban 60 mg orally once daily for 6 months.

### 2.3. Model Structure and Assumptions

**Figure 1:**
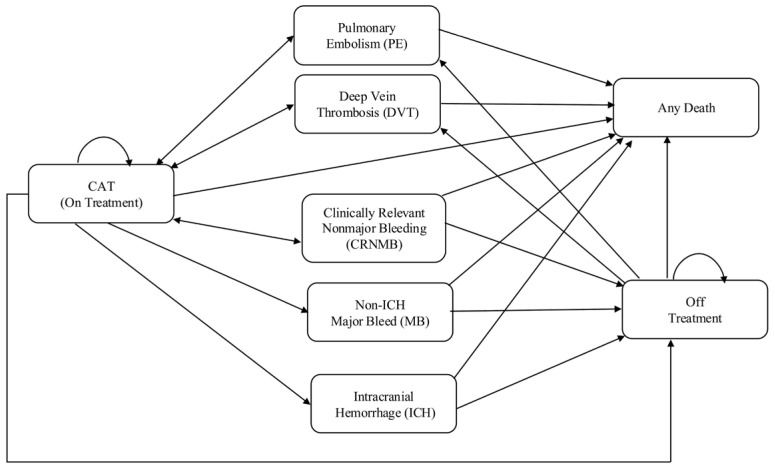
A schematic diagram for a Markov model. Abbreviations: CAT, cancer-associated thrombosis; PE, pulmonary embolism; DVT, deep venous thrombosis; CRNMB, clinically relevant nonmajor bleeding; MB, major bleeding; ICH, intracranial hemorrhage.

A state-transition Markov model with a one-month cycle length was employed in MS Excel365^®^. The model consisted of eight health states, including CAT on treatment, pulmonary embolism (PE), deep vein thrombosis (DVT), clinically relevant nonmajor bleeding (CRNMB), non-intracranial hemorrhage major bleeding (non-ICH MB), intracranial hemorrhage (ICH), off treatment, and any death (Figure 1). The model was adapted from a previous cost-effectiveness analysis [9]. Patients entered the model at the on-treatment health state. During the six-month treatment, patients could experience events such as PE, DVT, CRNMB, non-ICH MB, and ICH, or completed their treatment and moved to the off-treatment health state. Patients who experienced recurrent pulmonary embolism (rPE) or recurrent deep vein thrombosis(rDVT) could move back to on-treatment after the complete treatment of acute event, while patients who experienced non-ICH MB or ICH moved to off-treatment health state. Patients who experienced CRNMB could move to off-treatment or back to on-treatment. All health states could move to death health state.

Several assumptions were made. First, we assumed that patients who experienced rPE or rDVT or CRNMB received the same treatment after the completion of acute event. Second, patients with non-ICH MB or ICH were assumed to discontinue anticoagulants if they survived. Third, patients had higher risks for rVTE when off treatment than on treatment and that risk varied according to the time away from the initial index event. Fourth, cancer mortality was assumed to occur at a constant rate in all states. Lastly, we assumed that patients could experience adverse events during treatment, but no events would occur after treatment completion. Our model assumed no further VTE or bleeding after six months of therapy and after treatment discontinuation. Although patients with CAT remain at a somewhat increased risk beyond 6 months, evidence suggests that the excess risk diminishes substantially after the acute phase, and long-term event rates are low. The model assumed that patients who experienced rVTE or CRNMB continued on their initial anticoagulation regimen. In clinical practice, switching does occur, particularly after bleeding events in some patients; however, empirical data describing switching patterns in CAT are limited, and incorporating switching would require additional uncertain assumptions. Thus, we decide to assume that patients who experienced adverse events would remain in the same treatment which allows the model to not be excessively sophisticated and feasible.

### 2.4. Model Inputs

#### 2.4.1. Efficacy, Transitional Probabilities, and Mortality

The treatment effects of each DOAC compared with LMWH were retrieved from our systematic umbrella review with an updated network meta-analysis. A systematic umbrella review comparing DOACs with LMWH or warfarin in patients with CAT was conducted. A systematic review was searched from inception to 31 May 2024 in Cochrane Central, EMBASE, and PubMed. Randomized controlled trials found in the included systematic reviews were used in our network meta-analysis to estimate the treatment effects of DOACs compared with LMWH or warfarin. Full methodological details of the systematic umbrella review and network meta-analysis are provided in the Supplementary SI. The outcomes of interest included rVTE, MB, and CRNMB. Briefly, we identified 15 systematic reviews: 11 reviews compared DOACs to LMWH, and four reviews compared DOACs to warfarin. Our systematic umbrella review including 12 randomized controlled trials [17,18,19,20,21,22,23,24,25,26,27,28] showed that each DOAC had a trend indicating a lower risk of rVTE than LMWH. The risks of MB were not significantly higher but were significantly higher in CRNMB for each DOAC. The treatment effects and safety of anticoagulant therapy were assumed to be constant over time. The transitional probabilities of VTE patients receiving LMWH were based on previous studies [9]. All clinical inputs are presented in Table 1. The mortality rates among patients with CAT were based on previous studies [19,20] and the Thai age-specific mortality life table [29]. The cancer-related mortality was added to the Thai age-specific mortality for each health state, while the event-specific mortality (such as ICH, non-ICH MB, etc.) was also added the Thai age-specific mortality according to each event health state.

#### 2.4.2. Resource Use and Costs

According to the Thai health technology assessment guidelines [30], only direct medical and direct non-medical costs were included in this study. Indirect cost was excluded to avoid double-counting in economic evaluation using cost–utility study from a societal perspective. Drug prices and direct medical costs were obtained from a university-affiliated hospital’s electronic hospital database. Direct medical costs included costs for diagnostics, other medication costs, outpatient visits, and inpatient visits. Direct non-medical costs, including transportation, additional food and caregiver costs were obtained from the Thai Standard Cost Lists for health technology assessment [33]. All the costs and resources are listed in Table 1. Costs were adjusted for inflation rate to 2024 values using the consumer price index [35] and converted from Thai baht (THB) to US dollar (USD) using an average currency exchange rate of THB 32.788 per USD [36]. Hospital cost data were derived from hospital billing and charge databases. Where hospital charges were reported, these were converted to economic costs using cost-to-charge ratios (CCRs) [37]. The International classification of diseases version 10 (ICD-10) used to estimate costs of treatment is presented in Supplementary SII Table S4.

#### 2.4.3. Utilities

Utility weights and their corresponding 95% confidence intervals (CIs) were derived from a previously published study conducted on patients with CAT. The study included participants from North America, Asia, and Europe. The study used the EQ-5D-3L instrument to estimate the utility weights. Utility decrements associated with PE, DVT, and CRNMB bleeds were subtracted from baseline utility [34]. All utilities are listed in Table 1.

#### 2.4.4. Model Validation

An oncologist and a cardiology pharmacist clinically validated the model and its assumptions during the internal individual meeting. A health economist verified the model codes to ensure their validity.

#### 2.4.5. Analyses

The model outcomes included total lifetime costs and quality-adjusted life-years (QALYs) with a 3% annual discount rate. A scenario analysis was conducted by changing the price of DOACs from the median purchasing price to the median reference price provided by the drug and medical supply information center (DMSIC) [32]. The incremental cost-effectiveness ratio (ICER) per QALY gained was calculated. The willingness-to-pay (WTP) of THB 160,000/QALY (USD 4,879.83), was used as the cost-effectiveness threshold according to the Thai health technology assessment guidelines [30]. The robustness of the findings was assessed using one-way deterministic sensitivity analyses and probabilistic sensitivity analyses (PSAs). A series of one-way sensitivity analyses were performed to explore uncertainties among significant inputs by varying inputs using their 95% CI. The variations were assumed to be ±20 percent from the mean value. The distributions assumed for the input parameters were gamma (cost), beta (utility weights and TP), and lognormal (RR). We performed additional sensitivity and subgroup analyses based on relevant clinical scenarios. All data analyses were performed in Microsoft Excel for Mac as the tornado diagrams presented in Supplementary SIII Figure S3–S6. PSA was conducted using 10,000 iterations of Monte Carlo simulation to generate the cost-effectiveness analysis (CEA) plane. Each input was assigned a specific probability distribution based on its mean value, and the 95% credible interval.

### 2.5. Ethical Approval

The study protocol was approved by the Human Research Ethics Committee, Rajavithi Hospital, Thailand, 012/2567/E. As this study used retrospective, anonymized data extracted from the hospital database, the requirement for written informed consent was waived.

## 3. Results

### 3.1. Base-Case Analyses

**Figure 2:**
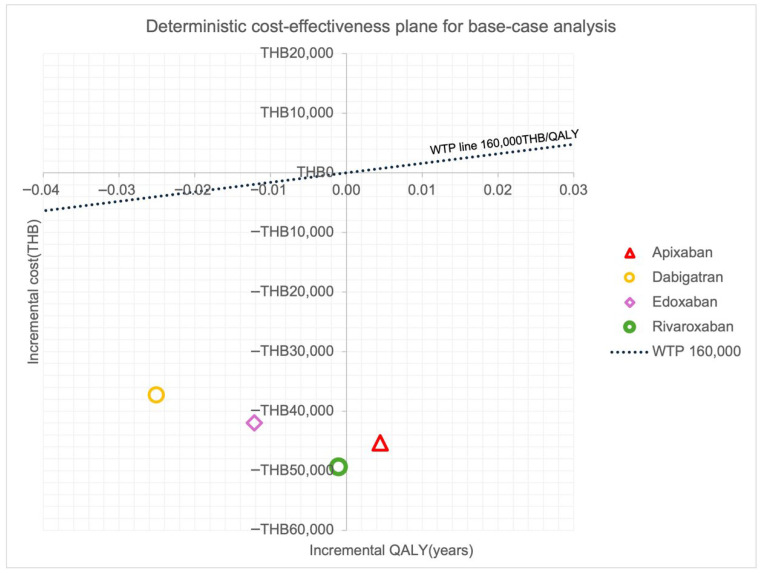
Base-case analysis findings. Abbreviations: THB, Thai baht; QALY, quality-adjusted life-year.

The total lifetime cost of LMWH was THB 70,928 (USD 2163) with the outcome of 0.771 QALYs. The total lifetime costs of apixaban, dabigatran, edoxaban, and rivaroxaban were THB 26,323 (USD 803), THB 33,667 (USD 1027), THB 29,570 (USD 902), and THB 22,310 (USD 680), respectively. The QALYs of apixaban, dabigatran, edoxaban, and rivaroxaban were 0.775, 0.746, 0.759, and 0.770 QALYs, respectively. Compared to LMWH, apixaban had slightly better life-years, QALYs, and reduced total costs. Apixaban provided 0.004 additional QALYs to LMWH with a decreased cost of THB 44,605 (USD 1360), resulting in a dominance option over LMWH. On the other hand, dabigatran, edoxaban, and rivaroxaban showed lower total costs and lower life-years and QALYs compared with LMWH (Table 2). On the other hand, dabigatran, edoxaban, and rivaroxaban resulted in lower total lifetime cost but reduced life-years and QALY compared to LMWH (Table 2). The cost-effectiveness planes for base-case analysis are presented in Figure 2.

A scenario analysis by changing the medication prices from DMSIC [32] indicated that apixaban remained cost-effective. The lifetime cost of apixaban was THB 17,914 lower than that of LMWH. Similarly, other DOACs were less costly and less effective than LMWH (Table 3).

### 3.2. Sensitivity Analyses

**Figure 3:**
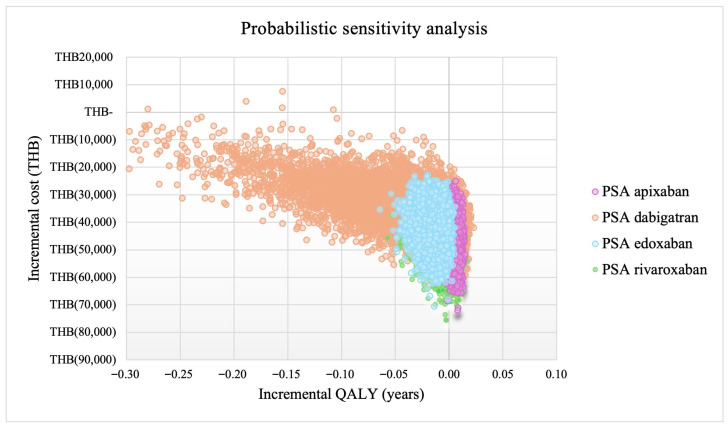
Probabilistic sensitivity analysis. Abbreviations: THB, Thai baht; QALY, quality-adjusted life-year.

One-way sensitivity analysis was performed and displayed as the tornado diagrams in Supplementary SIII Figure S3–S6. The analysis indicated that cost per cycle of LMWH and risk ratio of MB among patients with CAT were the most influential factors for both lifetime costs and QALYs. The findings of all DOACs were robust when examining all value ranges of each parameter. PSA supported this finding, showing that apixaban was cost-saving compared to LMWH. The probability of lower lifetime cost with higher QALY for apixaban was 85.9% while the probability of lower lifetime cost and QALY was 14.1%. In contrast, dabigatran, edoxaban, and rivaroxaban showed lower probabilities (14.63%, 1.35%, and 44.07%, respectively) in the right lower quadrant, reflecting substantial uncertainty and their lower effectiveness relative to LMWH. The PSA for each DOAC is presented in Figure 3 and Supplementary SIII Figure S7–S10.

## 4. Discussion

This study aimed to assess the cost-effectiveness of DOACs on VTE in patients with CAT in a Thai university-affiliated hospital. We found that apixaban was a dominant option compared with LMWH, whereas the other DOACs were less costly but also less effective. Apixaban yielded slightly higher QALYs and lower total costs than LMWH, indicating dominance. In contrast, dabigatran, edoxaban, and rivaroxaban produced marginal cost-savings but lower QALYs, indicating reduced clinical effectiveness compared with LMWH. However, despite their slightly lower QALYs, their substantially lower total costs, the uncertainty around those findings should be considered in the decision-making process.

Our sensitivity analysis showed the robustness of the main results. Our findings were inconsistent with previous cost–utility studies from the Netherlands, Brazil, and China [7,8,11], which indicated that DOACs were cost-effective compared with LMWH among patients with CAT. In contrast, studies from the United States [9,10] reported that DOACs reduced overall costs but did not improve QALY compared with LMWH. These discrepancies may be explained by several contextual factors. First, the cost structures in Thailand differ substantially from those in high-income and middle-income countries; drug prices, hospitalization fees, and healthcare resource usage are generally lower, which can influence the relative cost-effectiveness of LMWH and DOACs. Second, management pathways for CAT vary across settings. In Thailand, LMWH remains widely used as the standard treatment, and access to DOACs varies depending on reimbursement schemes, potentially affecting both costs and treatment outcomes compared with countries where DOACs are more uniformly adopted. Third, the differences in cancer epidemiology including the distribution of cancer types, stage at diagnosis, and baseline CAT risk may lead to variations in model inputs and treatment effects across studies.

We found that only apixaban could provide higher QALYs than LMWH, while the other DOACs provide lower QALYs than LMWH. This is because our systematic umbrella review and network meta-analysis indicated that apixaban could provide better clinical outcomes, including a decrease in the risks of rVTE, on average, while the other DOACs were associated with worse clinical outcomes, including higher risks of rVTE, MB, and CRNMB compared to LMWH. Our umbrella review results are similar to previous systematic review and meta-analysis which showed the better clinical outcomes in apixaban but not for other DOACs [18,22].

As our study was based on the DOAC prices from the purchasing price of a university-affiliated hospital, which were the DOAC originators, the prices might be different from the purchasing prices of other hospitals and might not represent the medication prices of DOACs for the whole country because there are generic drugs of each DOAC available in Thailand. Thus, we performed a scenario analysis using the median purchasing prices of each DOAC from the DMSIC [32]. We found that the median prices of each DOAC were lower than the purchasing price of the hospital resulting in the lower lifetime cost of each DOAC compared with LMWH. However, because the clinical outcomes are the same as the main analysis, the cost-effectiveness findings were unchanged: apixaban remained the only medication that was the dominant option for patients with CAT but not for other DOACs.

Our study did not consider the treatment cost of cancer and it was assumed to be equal for patients receiving either LMWH or DOACs. These could minimally affect the findings because patients are usually treated with the same treatments for cancer regardless of CAT. Thus, we believe that the assumption is still valid.

An important assumption was that patients who experienced non-ICH MB and ICH permanently discontinued anticoagulants. However, there might be some patients who survive from non-ICH MB or ICH still required anticoagulants. Thus, the assumptions might not be reflective of all patients with CAT; however, we believe that the assumption could still reflect the majority of patients with CAT who had non-ICH MB or ICH and mostly discontinued anticoagulants.

Our one-way sensitivity analysis showed that risk ratios of MB for each DOAC were the most influential factor for QALY. However, our systematic umbrella review and meta-analysis combined both ICH and non-ICH MB as a joint outcome. Accordingly, the model applied the same relative risk estimate to both ICH and non-ICH MB. Although ICH generally carries higher mortality risk and greater utility loss, disaggregating these events would have required additional assumptions. To overcome this limitation, event-specific costs and utility decrements were incorporated, and our probabilistic sensitivity analyses demonstrated that the overall model conclusions remained robust.

Our model was not stratified by cancer type, which might have influenced thrombotic and bleeding risks across different malignancies due to insufficient event-specific estimates for rVTE and bleeding across individual cancer subgroups. Future research should address this heterogeneity to improve the precision of cost-effectiveness estimates. However, we performed a PSA to explore the uncertainty around inputs and found that most iterations fell into the lower-right quadrant for apixaban, while most iterations fell into the lower-left quadrant for other DOACs. These indicated the robustness of our main findings.

The QALY differences observed between treatment strategies were small, these marginal gains may have limited clinical relevance. Decision makers may reasonably interpret these values as indicating broadly comparable health outcomes across DOACs and LMWH. Therefore, factors such as route of administration, convenience, patient preference, and acquisition costs may play a more influential role in real-world decision making procedures compared to the small numerical differences in QALYs alone. In addition, there were uncertainties around efficacy and safety inputs which might affect the cost-effective conclusions. The decision-making procedures might also consider these uncertainties in the decision process. Our limitations should be mentioned. First, an important limitation is that the treatment cost of CAT was derived from a single university-affiliated tertiary hospital in Bangkok, and the costs may vary across other healthcare settings in Thailand. However, because patients with CAT are typically managed in tertiary-care or teaching hospitals due to the complexity of the condition, the study setting is likely to be reasonably representative of similar high-level care hospital where most CAT patients receive treatment. Thus, our findings might be generalized to similar types of hospitals but the generalizability to other types of hospitals or national level should be performed with caution. Second, the similarity in life-years across strategies reflects the natural history of CAT. Evidence indicates that the risks of rVTE and MB are concentrated in the early treatment period (0–6 months) [38], during which mortality risk is also highest. Once the acute event has resolved and anticoagulation is discontinued, the risks of recurrence and bleeding decline substantially and approximate the baseline risk associated with underlying cancer. This pattern is well-documented in longitudinal CAT studies, which consistently show markedly lower recurrence and bleeding rates beyond the initial treatment phase [39]. Therefore, the limited long-term survival of patients with active cancer, combined with the short-term concentration of VTE related risks, explains why life-years cluster around one year even under a lifetime horizon. Nevertheless, sensitivity analyses were conducted to test the robustness of these assumptions, and the results remained consistent. Third, another important limitation was that our study did not consider the adjustment of LMWH and DOAC doses by other factors such as age, weight, renal function, or genetic variations. The dosages used were based on a standard recommendation from clinical guidelines and pivotal trials. A sensitivity analysis on alternative dosing was not performed due to limited evidence in CAT patients. In addition, our study aimed to assess the cost-effectiveness of DOACs for patients with CAT as a whole; therefore, these factors were not considered. Fourth, our model included patients aged 60 years or older and excluded warfarin as a comparator, which may limit the generalizability of the findings to younger populations or other anticoagulants. Finally, several model parameters, including clinical event probabilities and utilities were sourced from international studies due to the limited availability of Thai-specific data. Although such evidence is commonly used in economic evaluations in settings with constrained local data, variations in population characteristics, clinical practice patterns, and healthcare system structures may affect the findings. However, because of the limitations of country-specific data, we believe our approach was acceptable for the study’s context.

Our findings are important information for the hospital policymakers to consider whether some patients with CAT should be financially supported by the hospital healthcare fund because the listing of DOACs in the NLEM has been considered. In addition, our findings could be viewed as empirical evidence in national policy decision-making procedures considering whether DOAC should be listed in the NLEM; however, some uncertainties around the findings should also be considered during the decision-making process at both hospital and national levels.

## 5. Conclusions

This study revealed that apixaban is likely to be the preferred option for treating patients with CAT. However, policy decision-making procedures should take into account the uncertainties involved in the implementation of this practice.

## Figures and Tables

**Table 1:** Parameters for model input.

Parameter	Value	Range	Distribution	Source
Transition probabilities of patients receiving enoxaparin				
probability of rPE (month 1–6)	0.00775	0.0062–0.0093	beta	[9]
probability of rPE (month 7–12)	0.00185	0.0015–0.0022	beta	[9]
probability of rDVT (month 1–6)	0.00725	0.0058–0.0087	beta	[9]
probability of rDVT (month 7–12)	0.00425	0.0034–0.0051	beta	[9]
probability of CRNMB (month 1–6)	0.0118	0.0094–0.0142	beta	[9]
probability of MB (month 1–6)	0.0044	0.0035–0.0053	beta	[9]
probability of ICH (month 1–6)	0.0009	0.0007–0.0011	beta	[9]
probability of rPE when off drug (month 1–6)	0.02035	0.0163–0.0244	beta	[9]
probability of rPE when off drug (month 7–12)	0.0033	0.0026–0.004	beta	[9]
probability of rDVT when off drug (month 1–6)	0.019	0.0152–0.0228	beta	[9]
probability of rDVT when off drug (month 7–12)	0.0073	0.0058–0.0088	beta	[9]
probability of drug discontinuation	0.028	0.0224–0.0336	beta	[9]
probability of cancer death (month 1–6)	0.04515	0.0361–0.0542	beta	[9]
probability of cancer death (month 7–12)	0.0321	0.0257–0.0385	beta	[9]
probability of death due to PE	0.13515	0.1081–0.1622	beta	[9]
probability of death due to non-ICH MB	0.08695	0.0696–0.1043	beta	[9]
probability of death due to ICH	0.25	0.2–0.3	beta	[9]
Efficacy parameters of DOACs ^#^				
Risk of overall rVTE between DOACs and LMWH				
apixaban	0.57	0.3–1.1	lognormal	retrieved from our systematic umbrella review
edoxaban	0.68	0.45–1.04	lognormal	retrieved from our systematic umbrella review
dabigatran	0.95	0.23–3.97	lognormal	retrieved from our systematic umbrella review
rivaroxaban	0.68	0.38–1.21	lognormal	retrieved from our systematic umbrella review
Risk of MB between DOACs and LMWH				
apixaban	0.83	0.5–1.38	lognormal	retrieved from our systematic umbrella review
edoxaban	1.86	1.15–3.01	lognormal	retrieved from our systematic umbrella review
dabigatran	2.83	0.57–14.12	lognormal	retrieved from our systematic umbrella review
rivaroxaban	1.22	0.59–2.51	lognormal	retrieved from our systematic umbrella review
Risk of CRNMB between DOACs and LMWH				
apixaban	1.31	0.95–1.81	lognormal	retrieved from our systematic umbrella review
edoxaban	1.38	1.05–1.83	lognormal	retrieved from our systematic umbrella review
dabigatran	3.55	1.27–9.93	lognormal	retrieved from our systematic umbrella review
rivaroxaban	2.27	1.53–3.36	lognormal	retrieved from our systematic umbrella review
Fixed Assumptions				
Discount (%)	3		fixed	[30]
Age (years)	60		fixed	[12]
Weight (kilograms)	60		fixed	[31]
Direct medical treatment costs (THB)				
Cost estimates per cycle * (per month; THB)				
cost enoxaparin per month	12,198.00	9758.40–14,637.60	gamma	hospital database
apixaban cost for first cycle	3384.76	2707.81–4061.71	gamma	hospital database
edoxaban cost for first cycle	4413.75	3531.00–5296.50	gamma	hospital database
dabigatran cost for first cycle	4842.00	3873.60–5810.40	gamma	hospital database
rivaroxaban cost for first cycle	2341.41	1873.13–2809.69	gamma	hospital database
apixaban cost for following cycle	2744.40	2195.52–3293.28	gamma	hospital database
edoxaban cost for following cycle	2856.90	2285.52–3428.28	gamma	hospital database
dabigatran cost for following cycle	3370.80	2696.64–4044.96	gamma	hospital database
rivaroxaban cost for following cycle	1662.90	1330.32–1995.48	gamma	hospital database
apixaban cost for first cycle for sensitivity analysis	1233.89	987.11–1480.67	gamma	[32]
edoxaban cost for first cycle for sensitivity analysis	4165.25	3332.20–4998.30	gamma	[32]
dabigatran cost for first cycle for sensitivity analysis	4058.25	3246.60–4869.90	gamma	[32]
rivaroxaban cost for first cycle for sensitivity analysis	2294.46	1835.57–2753.35	gamma	[32]
apixaban cost for following cycle for sensitivity analysis	1000.45	800.36–1200.54	gamma	[32]
edoxaban cost for following cycle for sensitivity analysis	2856.90	2285.52–3428.28	gamma	[32]
dabigatran cost for following cycle for sensitivity analysis	2728.50	2182.80–3274.20	gamma	[32]
rivaroxaban cost for following cycle for sensitivity analysis	1506.39	1205.11–1807.67	gamma	[32]
Cost of treatment (THB)				
cost of PE treatment (IPD)	85,098.42	68,078.74–102,118.10	gamma	hospital database
cost of MB(ICH) treatment (IPD)	144,775.67	115,820.54–173,730.80	gamma	hospital database
cost of MB (non-ICH) treatment (IPD)	57,597.65	46,078.12–69,117.18	gamma	hospital database
cost of CRNMB treatment (OPD)	1936.69	1549.35–2324.03	gamma	hospital database
cost of DVT treatment (OPD)	2975.58	2380.46–3570.70	gamma	hospital database
Direct non-medical costs (THB)				
cost of travel	147.78	118.22–177.34	gamma	[33]
cost of food	71.64	57.31–85.97	gamma	[33]
cost of caregiver	104.14	83.32–124.97	gamma	[33]
Utilities				
utility of CAT	0.65	0.62–0.67	beta	[34]
utility of rVTE	0.57	0.49–0.64	beta	[34]
utility of MB	0.59	0.46–0.69	beta	[34]
utility of ICH	0.33	0.14–0.53	beta	[34]
utility of CRNMB	0.62	0.57–0.67	beta	[34]

Abbreviations: CAT, cancer-associated thrombosis; rVTE, recurrent venous thromboembolism; PE, pulmonary embolism; rPE, recurrent pulmonary embolism; DVT, deep venous thrombosis; rDVT, recurrent deep venous thrombosis; CRNMB, clinically relevant nonmajor bleeding; MB, major bleeding; ICH, intracranial hemorrhage; DOACs, direct oral anticoagulants; LMWH, low-molecular weight heparin; THB, Thai baht. ^#^ The treatment effects of each DOAC compared with LMWH were retrieved from our systematic umbrella review with an updated network meta-analysis. * Drug prices and direct medical costs were obtained from a university-affiliated hospital’s electronic hospital database.

**Table 2:** Base-case cost-effectiveness results (drug prices from Rajavithi hospital database).

Treatment	Cost (THB)	Cost (USD)	Life-Years	QALYs	Incremental Cost, THB	Incremental Cost, USD	Incremental QALY	ICER (95% CI) THB/QALY
enoxaparin	70,928	2163	1.198	0.771	reference			
apixaban	26,323	803	1.204	0.775	−44,605	−1360	0.004	cost-saving (dominance)
dabigatran	33,667	1027	1.160	0.746	−37,261	−1136	−0.025	less costly and less effective
edoxaban	29,570	902	1.179	0.759	−41,358	−1261	−0.012	less costly and less effective
rivaroxaban	22,310	680	1.196	0.770	−48,618	−1483	−0.001	less costly and less effective

Abbreviations: THB, Thai baht; USD, US dollars (USD 32.79 per Thai baht); QALY, quality-adjusted life-year; ICER, incremental cost-effectiveness ratio; CI, confidence interval.

**Table 3:** Base-case cost-effectiveness results (drug prices from DMSIC).

Treatment	Cost (THB)	Cost (USD)	Life-Years	QALYs	Incremental Cost, THB	Incremental Cost, USD	Incremental QALY	ICER (95% CI) THB/QALY
enoxaparin	70,928	2163	1.198	0.771	reference			
apixaban	17,914	546	1.204	0.775	−53,014	−1617	0.004	cost-saving (dominance)
dabigatran	30,783	939	1.160	0.746	−40,145	−1224	−0.025	less costly and less effective
edoxaban	29,378	896	1.179	0.759	−41,550	−1267	−0.012	less costly and less effective
rivaroxaban	21,699	662	1.196	0.770	−49,229	−1501	−0.001	less costly and less effective

Abbreviations: THB, Thai baht; USD, US dollars (32.79 USD per Thai baht); QALY, quality-adjusted life-year; ICER, incremental cost-effectiveness ratio; DMSIC, drug and medical supply information center, Ministry of Public Health; CI, confidence interval.

## Data Availability

Restrictions apply to the availability of these data. The data that support the findings of this study are available from the authors upon reasonable request and with permission from the Human Research Ethics Committee of Rajavithi Hospital. The formal data sharing agreement with the research team must also be performed. The data are not publicly available due to privacy and ethical restrictions.

## Supplementary Materials

The following supporting information can be downloaded at https://www.mdpi.com/article/10.3390/jcm15010212/s1, Supplementary Materials SI; Table S1: Detailed search strategy; Table S2: Table of overlapping RCTs from systematic reviews and meta-analysis; Table S3: The AMSTAR 2 quality assessment of studies included in the umbrella review; Figure S1: The Preferred Reporting Items for Systematic Reviews and Meta-analyses (PRISMA) flow diagram of study selection; Figure S2: Risk of Bias of included RCTs; Full methodological details of the systematic umbrella review and network meta-analysis, Supplementary Materials SII; Table S4: Diagnosis codes used to identify the VTE and bleeding events, Supplementary Materials SIII; Figure S3: Tornado diagram for deterministic sensitivity analyses for incremental cost and QALY of apixaban compare to LMWH. Figure S4: Tornado diagram for deterministic sensitivity analyses for incremental cost and QALY of dabigatran compared to LMWH. Figure S5: Tornado diagram for deterministic sensitivity analyses for incremental cost and QALY of edoxaban compared to LMWH. Figure S6 Tornado diagram for deterministic sensitivity analyses for incremental cost and QALY of rivaroxaban compared to LMWH. Figure S7: Probabilistic cost-effectiveness plane results of apixaban compared to LMWH. Figure S8: Probabilistic cost-effectiveness plane results of dabigatran compared to LMWH. Figure S9: Probabilistic cost-effectiveness plane results of edoxaban compared to LMWH. Figure S10: Probabilistic cost-effectiveness plane results of rivaroxaban compared to LMWH. References [40,41,42,43,44,45,46,47,48,49,50,51,52,53,54,55,56,57,58,59] are cited in Supplementary Materials
