# Supplementary material for: Economic Evaluation of Direct Oral Anticoagulants Versus Low-Molecular Weight Heparin for Cancer-Associated Thrombosis in a Thai University-Affiliated Hospital
Source: J Clin Med. 2025 Dec 27;15(1):212. doi: 10.3390/jcm15010212 (PMC12786408; doi:10.3390/jcm15010212)
Supplement: Supplementary file 1 [file jcm-15-00212-s001.zip › jcm-4023962-supplementary.pdf]

Supplementary I

Methodological details of the systematic umbrella review and network meta-analysis

Methods

A 2-step review process were undertaken. First, a systematic umbrella review of current systematic reviews was conducted to determine the current evidence of anticoagulants among patients with CAT. The evidence was summarized descriptively. Second, RCTs found in each systematic review was evaluated to identify relevant RCTs and then systematic umbrella review were performed

Search strategy

A comprehensive literature search was performed in the Cochrane CENTRAL, EMBASE, and PubMed from their inception to May 31, 2024. Further studies were identified by manually searching the bibliographies of retrieved articles. The search terms included: “cancer” OR “tumor” OR “neoplasms” OR “malignancy” AND “anticoagulants” OR “factor Xa inhibitors” OR “heparin” OR “dabigatran” OR “rivaroxaban” OR “edoxaban” OR “apixaban” OR “heparin, low-molecular-weight” AND “venous thromboembolism” OR “pulmonary embolism” OR “venous thrombosis” AND "systematic review" OR “meta-analysis” The detailed search strategy is provided in Supplementary I Table 1.

Table S1 Detailed search strategy

#	Search Syntax	
**PubMed (n= 15)**		
	Search: **#1 AND #2 AND #4 AND #5 AND #6 AND #7 AND #8** Filters: **Full text, Meta-Analysis, Systematic Review, in the last 10 years**<br>((((("cancer"[Text Word]) OR ("malignancy"[Text Word])) OR ("neoplasms"[MeSH Terms])) AND ((y_10[Filter]) AND (meta-analysis[Filter] OR systematicreview[Filter]) AND (fft[Filter]))) AND ((((((((((("Direct-Acting Oral Anticoagulant"[Text Word]) OR	

#	Search Syntax	
	("Dabigatran"[Text Word])) OR ("Endoxaban"[Text Word])) OR ("Rivaroxaban"[Text Word])) OR ("Apixaban"[Text Word])) OR ("Factor Xa Inhibitor"[MeSH Terms])) OR ("Direct-Acting Oral Anticoagulant"[MeSH Terms])) OR ("Dabigatran"[MeSH Terms])) OR ("Endoxaban"[MeSH Terms])) OR ("Rivaroxaban"[MeSH Terms])) OR ("Apixaban"[MeSH Terms])) AND ((y_10[Filter]) AND (meta-analysis[Filter] OR systematicreview[Filter]) AND (fft[Filter]))) AND (((("low molecular weight heparin"[Text Word]) OR ("enoxaparin"[Text Word])) OR ("low molecular weight heparin"[MeSH Terms])) AND ((y_10[Filter]) AND (meta-analysis[Filter] OR systematicreview[Filter]) AND (fft[Filter]))) AND ((((("deep venous thromboses"[Text Word]) OR ("venous thromboembolism"[Text Word])) OR ("venous thrombosis"[MeSH Terms])) OR ("venous thromboembolism"[MeSH Terms])) AND ((y_10[Filter]) AND (meta-analysis[Filter] OR systematicreview[Filter]) AND (fft[Filter]))) AND ((((("pulmonary embolism"[Text Word]) OR ("thromboembolism"[Text Word])) OR ("pulmonary embolism"[MeSH Terms])) OR ("thromboembolism"[MeSH Terms])) AND ((y_10[Filter]) AND (meta-analysis[Filter] OR systematicreview[Filter]) AND (fft[Filter]))) AND ((((("hemorrhage"[Text Word]) OR ("Bleeding"[Text Word])) OR ("Bleeding"[MeSH Terms])) OR ("hemorrhage"[MeSH Terms])) AND ((y_10[Filter]) AND (meta-analysis[Filter] OR systematicreview[Filter]) AND (fft[Filter]))) AND (((((("Mortality"[MeSH Terms])) OR ("Death"[MeSH Terms])) OR ("Death"[Text Word])) OR ("Mortality"[Text Word])) AND ((y_10[Filter]) AND (meta-analysis[Filter] OR systematicreview[Filter]) AND	

#	Search Syntax	
	(fft[Filter])))) AND ((y_10[Filter]) AND (meta-analysis[Filter] OR systematicreview[Filter]) AND (fft[Filter]))	
**EMBASE : OVID search(n= 27)**		
1	"cancer".mp. [mp=title, book title, abstract, original title, name of substance word, subject heading word, floating sub-heading word, keyword heading word, organism supplementary concept word, protocol supplementary concept word, rare disease supplementary concept word, unique identifier, synonyms, population supplementary concept word, anatomy supplementary concept word]	2,338,610
2	"malignancy".mp. [mp=title, book title, abstract, original title, name of substance word, subject heading word, floating sub-heading word, keyword heading word, organism supplementary concept word, protocol supplementary concept word, rare disease supplementary concept word, unique identifier, synonyms, population supplementary concept word, anatomy supplementary concept word]	191,371
3	"neoplasms".mp. [mp=title, book title, abstract, original title, name of substance word, subject heading word, floating sub-heading word, keyword heading word, organism supplementary concept word, protocol supplementary concept word, rare disease supplementary concept word, unique identifier, synonyms, population supplementary concept word, anatomy supplementary concept word]	3,234,997
4	Neoplasms/	524,112
5	1 or 2 or 3 or 4	4,028,527

#	**Search Syntax**	
6	"Direct-Acting Oral Anticoagulant".mp. [mp=title, book title, abstract, original title, name of substance word, subject heading word, floating sub-heading word, keyword heading word, organism supplementary concept word, protocol supplementary concept word, rare disease supplementary concept word, unique identifier, synonyms, population supplementary concept word, anatomy supplementary concept word]	180
7	"Dabigatran".mp. [mp=title, book title, abstract, original title, name of substance word, subject heading word, floating sub-heading word, keyword heading word, organism supplementary concept word, protocol supplementary concept word, rare disease supplementary concept word, unique identifier, synonyms, population supplementary concept word, anatomy supplementary concept word]	6,894
8	"Edoxaban".mp. [mp=title, book title, abstract, original title, name of substance word, subject heading word, floating sub-heading word, keyword heading word, organism supplementary concept word, protocol supplementary concept word, rare disease supplementary concept word, unique identifier, synonyms, population supplementary concept word, anatomy supplementary concept word]	2,431
9	"Rivaroxaban".mp. [mp=title, book title, abstract, original title, name of substance word, subject heading word, floating sub-heading word, keyword heading word, organism supplementary concept word, protocol supplementary concept word, rare disease supplementary concept word, unique identifier, synonyms, population supplementary concept word, anatomy supplementary concept word]	8,788

#	Search Syntax	
10	"Apixaban".mp. [mp=title, book title, abstract, original title, name of substance word, subject heading word, floating sub-heading word, keyword heading word, organism supplementary concept word, protocol supplementary concept word, rare disease supplementary concept word, unique identifier, synonyms, population supplementary concept word, anatomy supplementary concept word]	5,959
11	Factor Xa Inhibitors/	6,328
12	6 or 7 or 8 or 9 or 10 or 11	17,641
13	"low molecular weight heparin".mp. [mp=title, book title, abstract, original title, name of substance word, subject heading word, floating sub-heading word, keyword heading word, organism supplementary concept word, protocol supplementary concept word, rare disease supplementary concept word, unique identifier, synonyms, population supplementary concept word, anatomy supplementary concept word]	12,718
14	"heparin".mp. [mp=title, book title, abstract, original title, name of substance word, subject heading word, floating sub-heading word, keyword heading word, organism supplementary concept word, protocol supplementary concept word, rare disease supplementary concept word, unique identifier, synonyms, population supplementary concept word, anatomy supplementary concept word]	107,010
15	"enoxaparin".mp. [mp=title, book title, abstract, original title, name of substance word, subject heading word, floating sub-heading word, keyword heading word, organism supplementary concept word, protocol supplementary concept word, rare disease supplementary concept word,	6,649

#	Search Syntax	
	unique identifier, synonyms, population supplementary concept word, anatomy supplementary concept word]	
16	"dalteparin".mp. [mp=title, book title, abstract, original title, name of substance word, subject heading word, floating sub-heading word, keyword heading word, organism supplementary concept word, protocol supplementary concept word, rare disease supplementary concept word, unique identifier, synonyms, population supplementary concept word, anatomy supplementary concept word]	1,467
17	"low molecular weight heparin".mp. or Heparin, Low-Molecular-Weight/	16,873
18	13 or 14 or 15 or 16 or 17	110,545
19	"deep venous thromboses".mp. [mp=title, book title, abstract, original title, name of substance word, subject heading word, floating sub-heading word, keyword heading word, organism supplementary concept word, protocol supplementary concept word, rare disease supplementary concept word, unique identifier, synonyms, population supplementary concept word, anatomy supplementary concept word]	416
20	"venous thromboembolism".mp. [mp=title, book title, abstract, original title, name of substance word, subject heading word, floating sub-heading word, keyword heading word, organism supplementary concept word, protocol supplementary concept word, rare disease supplementary concept word, unique identifier, synonyms, population supplementary concept word, anatomy supplementary concept word]	33,601
21	"pulmonary embolism".mp. [mp=title, book title, abstract, original title, name of substance word, subject heading word, floating sub-heading	63,935

#	Search Syntax	
	word, keyword heading word, organism supplementary concept word, protocol supplementary concept word, rare disease supplementary concept word, unique identifier, synonyms, population supplementary concept word, anatomy supplementary concept word]	
22	"thromboembolism".mp. [mp=title, book title, abstract, original title, name of substance word, subject heading word, floating sub-heading word, keyword heading word, organism supplementary concept word, protocol supplementary concept word, rare disease supplementary concept word, unique identifier, synonyms, population supplementary concept word, anatomy supplementary concept word]	71,059
23	"deep venous thromboses".mp. or Venous Thrombosis/	30,770
24	19 or 20 or 21 or 22 or 23	138,000
25	"hemorrhage".mp. [mp=title, book title, abstract, original title, name of substance word, subject heading word, floating sub-heading word, keyword heading word, organism supplementary concept word, protocol supplementary concept word, rare disease supplementary concept word, unique identifier, synonyms, population supplementary concept word, anatomy supplementary concept word]	336,809
26	"Bleeding".mp. [mp=title, book title, abstract, original title, name of substance word, subject heading word, floating sub-heading word, keyword heading word, organism supplementary concept word, protocol supplementary concept word, rare disease supplementary concept word, unique identifier, synonyms, population supplementary concept word, anatomy supplementary concept word]	258,401

#	**Search Syntax**	
27	Hemorrhage/	84,837
28	25 or 26 or 27	503,856
29	"Mortality".mp. [mp=title, book title, abstract, original title, name of substance word, subject heading word, floating sub-heading word, keyword heading word, organism supplementary concept word, protocol supplementary concept word, rare disease supplementary concept word, unique identifier, synonyms, population supplementary concept word, anatomy supplementary concept word]	1,475,552
30	"Death".mp. [mp=title, book title, abstract, original title, name of substance word, subject heading word, floating sub-heading word, keyword heading word, organism supplementary concept word, protocol supplementary concept word, rare disease supplementary concept word, unique identifier, synonyms, population supplementary concept word, anatomy supplementary concept word]	1,033,283
31	Death/	20,852
32	29 or 30 or 31	2,230,054
33	5 and 12 and 18 and 24 and 28 and 32	122
34	"systematic review".mp. or "Systematic Review"/	353,198
35	"meta analysis".mp. or Meta-Analysis/	311,870
36	34 or 35	494,085
37	33 and 36	27
**CENTRAL (n= 10)**		

#	**Search Syntax**	
#1	("cancer"):ti,ab,kw OR ("malignancy"):ti,ab,kw OR ("neoplasms"):ti,ab,kw (Word variations have been searched)	249353
#2	("Direct-Acting Oral Anticoagulant"):ti,ab,kw OR (“Dabigatran”):ti,ab,kw OR (“Edoxaban”):ti,ab,kw OR (“Rivaroxaban”):ti,ab,kw OR (“Apixaban”):ti,ab,kw (Word variations have been searched)	4602
#3	("low molecular weight heparin"):ti,ab,kw OR (“enoxaparin”):ti,ab,kw OR (“heparin”):ti,ab,kw OR (“dalteparin”):ti,ab,kw (Word variations have been searched)	14990
#4	("deep venous thromboses"):ti,ab,kw OR ("venous thrombosis"):ti,ab,kw OR ("venous thromboembolism"):ti,ab,kw OR ("pulmonary embolism"):ti,ab,kw OR ("thromboembolism"):ti,ab,kw (Word variations have been searched)	17449
#5	("hemorrhage"):ti,ab,kw OR ("Bleeding"):ti,ab,kw (Word variations have been searched)	86722
#6	("Mortality"):ti,ab,kw OR ("Death"):ti,ab,kw (Word variations have been searched)	183496
#7	#1 AND #2 AND #3 AND #4 AND #5 AND #6	91
#8	("systematic review"):ti,ab,kw OR ("metaanalysis"):ti,ab,kw (Word variations have been searched)	21977
#9	#7 AND #8	10

Search results the systematic umbrella review of previous systematic reviews

A total of 21 systematic reviews were evaluated. Of those, 15 studies[1-15] met the inclusion criteria and were included. Six studies were excluded for the following reasons: (1) four studies had inadequate outcomes of interest[16-19]; and (2) two studies did not involve the patient population of interest.[29,30] shown in Supplementary I Figure 1

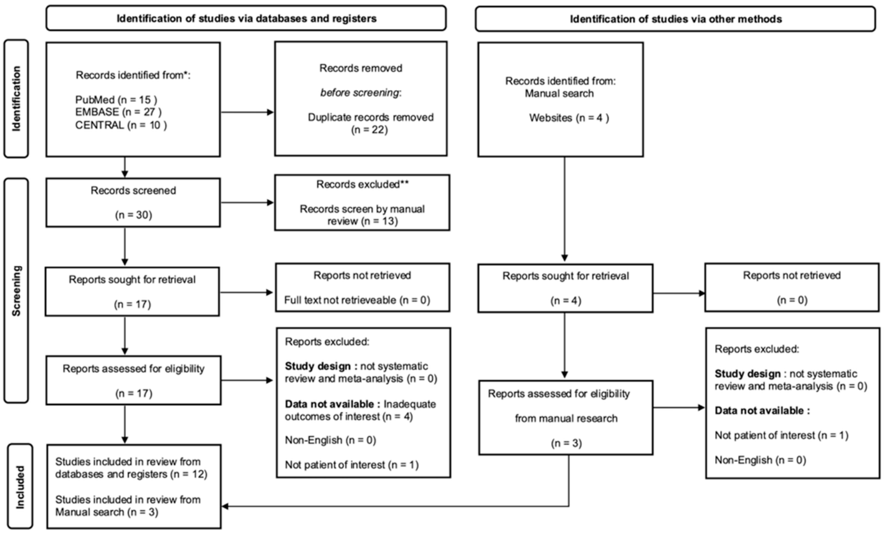

Figure S1 The Preferred Reporting Items for Systematic Reviews and Meta-analyses (PRISMA) flow diagram of study selection

Systematic reviews which were conducted to evaluate efficacy or safety of anticoagulants among patients with CAT were eligible for this systematic umbrella review. The inclusion criteria were developed based on four key components as follows:

P (Population): patients with CAT

I (Intervention): treatment with DOACs, including apixaban, edoxaban, dabigatran, or rivaroxaban

C (Comparison): treatment with LMWHs, including enoxaparin or dalteparin, or warfarin

O (Outcome): rVTE or major bleeding (MB), or clinically relevant nonmajor bleeding (CRNMB)

According to the framework abovementioned, the inclusion criteria were;

(1) systematic reviews involved patients with CAT

(2) systematic reviews compared among of DOACs, LMWHs, or warfarin

(3) systematic reviews which included RCTs and

(4) systematic reviews reported efficacy outcomes, including rPE, rVTE, cancer related VTE mortality, and all-cause mortality, as well as safety outcomes related to MB or CRNMB.

Study selection and eligibility criteria of RCTs identified in previous systematic reviews

We identified RCTs from the included systematic reviews. Studies were included if they met the following criteria; (1) studies were RCTs, (2) studies were conducted in patients with CAT and received rVTE treatment, (3) studies compared DOACs with LMWH or warfarin, and (4) studies reported at least one of the following outcomes as rVTE, MB, or CRNMB are presented in Supplementary I Table 2.

Table S2 Table of overlapping RCTs from systematic reviews and meta-analysis

No	Randomized Controlled Trials	Drugs: Intervention	Drugs: Control	Systematic Reviews and Meta-analysis from flow diagram of study selection<br>1<br>Alexandra C. Murphy et	2<br>Corinne Frere et al,2022[10]	3<br>Frits I. Mulder et al,2020[8]	4<br>Gustavo Muc oucah Sampaio Brandao	5<br>Haoyu Ning et al,2022[6]	6<br>Hiroki Ueyama et al,2020[3]	7<br>Irbaz Bin Riaz et al,2022[5]	8<br>Michela Giustozzi et al,2022[15]	9<br>Natale Daniele Brunetti et	10<br>Ruchi Desai et al,2020[12]	11<br>Shuyi Wu et al.,2022[1]	12<br>V. Mai et al,2020[9]	13<br>Xiaojun Song et al,2020[4]	14<br>Ying Dong et al,2019[11]	15<br>Yunsong Wang et al.,2019[2]
1	Agnelli et al.,2015 (AMPLIFY) [20]	Apixaban	Bridging*	X	X	X	X	X	/	X	X	X	/	/	X	X	X	/
2	Agnelli et al.,2020 (CARAVAGGIO)[21]	Apixaban	Dalteparin<br>Enoxaparin	/	/	/	/	/	/	/	/	X	/	/	X	/	X	X
3	Jwa Hoon Kim et al.,2022 [22]	Rivaroxaban or Apixaban	Dalteparin	X	X	X	X	/	X	X	X	X	X	X	X	X	X	X
4	McBane II et al.,2020 (ADAM-VTE) [23]	Apixaban	Dalteparin	/	/	/	/	/	/	/	/	/	/	/	/	/	X	X

No	Randomized Controlled Trials	Drugs (Intervention / Control)	Systematic Reviews and Meta-analysis from flow diagram of study selection														
			1	2	3	4	5	6	7	8	9	10	11	12	13	14	15
			Alexandra C. Murphy et	Corinne Frere et al,2022[10]	Frits I. Mulder et al,2020[8]	Gustavo Muc oucah Sampaio Brandao	Haoyu Ning et al,2022[6]	Hiroki Ueyama et al,2020[3]	Irbaz Bin Riaz et al,2022[5]	Michela Giustozzi et al,2022[15]	Natale Daniele Brunetti et	Ruchi Desai et al,2020[12]	Shuyi Wu et al.,2022[1]	V. Mai et al,2020[9]	Xiaojun Song et al,2020[4]	Ying Dong et al,2019[11]	Yunsong Wang et al.,2019[2]
5	Mokadem et al.,2020 [24]	Apixaban<br>Enoxaparin	X	X	X	X	/	X	X	X	X	X	/	X	X	X	X
6	Planquette et al.,2021 (CASTA-DIVA) [25]	Rivaroxaban<br>Dalteparin	X	/	X	X	/	X	X	X	X	X	/	X	X	X	X
7	Prins et al.,2013 (EINSTEIN pooled)[26,27]	Rivaroxaban<br>Bridging*	X	X	X	X	X	/ [26]	X	X	X	/ [27]	/ [27]	X	X	X	/ [26]
8	Raskob et al.,2016 (HOKUSAI-VTE)[28]	Edoxaban<br>warfarin	X	X	X	X	X	/	X	X	X	X	/	X	X	X	/

No	Randomized Controlled Trials	Drugs: Intervention<br>Control	1<br>Alexandra C. Murphy et	2<br>Corinne Frere et al,2022[10]	3<br>Frits I. Mulder et al,2020[8]	4<br>Gustavo Muc oucah Sampaio Brandao	5<br>Haoyu Ning et al,2022[6]	6<br>Hiroki Ueyama et al,2020[3]	7<br>Irbaz Bin Riaz et al,2022[5]	8<br>Michela Giustozzi et al,2022[15]	9<br>Natale Daniele Brunetti et	10<br>Ruchi Desai et al,2020[12]	11<br>Shuyi Wu et al.,2022[1]	12<br>V. Mai et al,2020[9]	13<br>Xiaojun Song et al,2020[4]	14<br>Ying Dong et al,2019[11]	15<br>Yunsong Wang et al.,2019[2]
9	Raskob et al.,2018 (HOKUSAI-VTE CANCER) [29]	Edoxaban<br>Dalteparin	/	/	/	/	/	/	/	/	/	/	/	/	/	/	/
10	Schrag et al.,2023 (CANVAS) [30]	All DOACs<br>Dalteparin	X	/	X	X	X	X	X	X	X	X	X	X	X	X	X
11	Schulman et al.,2015 (RECOVER I & II) [31-33]	Dabigatran<br>warfarin	X	X	X	X	X	/	X	X	X	/[32,33]	/[33]	X	X	X	/
12	Young et al.,2018 (SELECT-D) [34]	Rivaroxaban<br>Dalteparin	/	/	/	/	/	/	/	/	/	/	/	/	/	/	/

Systematic Reviews and Meta-analysis from flow diagram of study selection (columns 1–15)

Network meta-analysis findings for rVTE

Our network meta-analysis indicated that DOACs could reduce the risk of rVTE compared to LMWH (RR 0.66, 95%CI; 0.52 to 0.83) and warfarin (RR 0.54, 95% CI; 0.36 to 0.80). For each individual DOAC, no individual DOAC, including apixaban, edoxaban, rivaroxaban, or dabigatran, was different to LWMH in terms of the risk of rVTE. However, we found that apixaban (RR 0.45, 95%CI; 0.22 to 0.93) and edoxaban (RR 0.54, 95%CI; 0.32 to 0.90) had a lower risk of rVTE compared to warfarin.

Network meta-analysis findings for MB and CRNMB

Our network meta-analysis indicated that DOACs did not have a higher risk of MB compared to LMWH (1.22, 95%CI; 0.85 to 1.75) and warfarin (0.68, 95%CI; 0.39 to 1.17) (Table S4b). However, we found that DOACs had a higher risk of CRNMB compared to LMWH (1.57, 95%CI; 1.17 to 2.09), while DOACs did not have a higher risk of CRNMB compared to warfarin (0.76, 95%CI; 0.56 to 1.03). For each individual DOAC, edoxaban had a higher risk of MB compared to LMWH (1.86, 95%CI; 1.15 to 3.01) but not for other DOACs. We also found that dabigatran, edoxaban, and rivaroxaban had higher risks of CRNMB compared to LMWH but not for apixaban. All network meta-analysis for MB and CRNMB found no inconsistency.

Quality assessment of previous systematic reviews

Overall, most systematic reviews covered the important domains that were adhered to the AMSTAR-2 criteria including having a clearly defined PICO structure, comprehensive literature searches, and data were independently selected and extracted by two authors. However, some systematic reviews were lacking in certain domains including the lack of sufficient publication bias test, the mention of potential conflicts of interest and funding sources, a suitable procedure to assess the risk of bias, and the mentions of heterogeneity. The detailed quality assessment of the included systematic reviews using AMSTAR-2 are presented in Supplementary I Table S3.

Table S3 The AMSTAR 2 quality assessment of studies included in the umbrella review

**No**	**Content of the domain AMSTAR 2**	**Systematic Reviews and Meta-analysis from flow diagram of study selection**														
		**1**	**2**	**3**	**4**	**5**	**6**	**7**	**8**	**9**	**10**	**11**	**12**	**13**	**14**	**15**
		**Alexandra C. Murphy et al.,2022[7]**	**Corinne Frere et al,2022[10]**	**Frits I. Mulder et al,2020[8]**	**Gustavo Muc oucah Sampaio Brandao**	**Haoyu Ning et al,2022[6]**	**Hiroki Ueyama et al,2020[3]**	**Irbaz Bin Riaz et al,2022[5]**	**Michela Giustozzi et al,2022[15]**	**Natale Daniele Brunetti et al,2019[13]**	**Ruchi Desai et al,2020[12]**	**Shuyi Wu et al.,2022[1]**	**V. Mai et al,2020[9]**	**Xiaojun Song et al,2020[4]**	**Ying Dong et al,2019[11]**	**Yunsong Wang et al.,2019[2]**
1	Did the research questions and inclusion criteria for the review include the components of PICO?	Y	Y	Y	Y	Y	Y	Y	Y	Y	Y	Y	Y	Y	Y	Y
2	Did the report of the review contain an explicit statement that the review methods were established prior to the conduct of the review and did the report justify any significant	**Y**	**Y**	**Y**	**Y**	**Y**	**Y**	**Y**	**Y**	**Y**	**Y**	**Y**	**Y**	**Y**	**Y**	**Y**

No	Content of the domain AMSTAR 2	Systematic Reviews and Meta-analysis from flow diagram of study selection														
		**1**	**2**	**3**	**4**	**5**	**6**	**7**	**8**	**9**	**10**	**11**	**12**	**13**	**14**	**15**
		**Alexandra C. Murphy et al.,2022**[7]	**Corinne Frere et al,2022**[10]	**Frits I. Mulder et al,2020**[8]	**Gustavo Mucoucah Sampaio Brandao**	**Haoyu Ning et al,2022**[6]	**Hiroki Ueyama et al,2020**[3]	**Irbaz Bin Riaz et al,2022**[5]	**Michela Giustozzi et al,2022**[15]	**Natale Daniele Brunetti et al,2019**[13]	**Ruchi Desai et al,2020**[12]	**Shuyi Wu et al.,2022**[1]	**V. Mai et al,2020**[9]	**Xiaojun Song et al,2020**[4]	**Ying Dong et al,2019**[11]	**Yunsong Wang et al.,2019**[2]
	deviations from the protocol?															
3	Did the review authors explain their selection of the study designs for inclusion in the review?	Y	Y	Y	Y	Y	Y	Y	Y	Y	Y	Y	Y	N	N	N
4	Did the review authors use a comprehensive literature search strategy?	**PY**	**Y**	**Y**	**Y**	**Y**	**Y**	**Y**	**Y**	**Y**	**Y**	**Y**	**Y**	**Y**	**Y**	**Y**
5	Did the review authors perform study selection in duplicate?	Y	Y	Y	Y	Y	Y	Y	Y	Y	Y	Y	Y	Y	Y	Y

No	Content of the domain AMSTAR 2	Systematic Reviews and Meta-analysis from flow diagram of study selection														
		**1**	**2**	**3**	**4**	**5**	**6**	**7**	**8**	**9**	**10**	**11**	**12**	**13**	**14**	**15**
		**Alexandra C. Murphy et al.,2022[7]**	**Corinne Frere et al,2022[10]**	**Frits I. Mulder et al,2020[8]**	**Gustavo Muc oucah Sampaio Brandao**	**Haoyu Ning et al,2022[6]**	**Hiroki Ueyama et al,2020[3]**	**Irbaz Bin Riaz et al,2022[5]**	**Michela Giustozzi et al,2022[15]**	**Natale Daniele Brunetti et al,2019[13]**	**Ruchi Desai et al,2020[12]**	**Shuyi Wu et al.,2022[1]**	**V. Mai et al,2020[9]**	**Xiaojun Song et al,2020[4]**	**Ying Dong et al,2019[11]**	**Yunsong Wang et al.,2019[2]**
6	Did the review authors perform data extraction in duplicate?	Y	Y	Y	Y	Y	Y	Y	Y	Y	Y	Y	Y	Y	Y	Y
7	Did the review authors provide a list of excluded studies and justify the exclusions?	**Y**	**PY**	**Y**	**Y**	**Y**	**Y**	**Y**	**Y**	**Y**	**Y**	**Y**	**Y**	**Y**	**Y**	**Y**
8	Did the review authors describe the included studies in adequate detail?	Y	Y	PY	Y	Y	Y	Y	Y	PY	PY	Y	Y	Y	PY	PY
9	Did the review authors use a satisfactory technique for	**Y**	**Y**	**Y**	**Y**	**Y**	**Y**	**Y**	**Y**	**Y**	**Y**	**Y**	**Y**	**Y**	**Y**	**Y**

No	Content of the domain AMSTAR 2	Systematic Reviews and Meta-analysis from flow diagram of study selection														
		1	2	3	4	5	6	7	8	9	10	11	12	13	14	15
		Alexandra C. Murphy et al.,2022[7]	Corinne Frere et al,2022[10]	Frits I. Mulder et al,2020[8]	Gustavo Muc oucah Sampaio Brandao	Haoyu Ning et al,2022[6]	Hiroki Ueyama et al,2020[3]	Irbaz Bin Riaz et al,2022[5]	Michela Giustozzi et al,2022[15]	Natale Daniele Brunetti et al,2019[13]	Ruchi Desai et al,2020[12]	Shuyi Wu et al.,2022[1]	V. Mai et al,2020[9]	Xiaojun Song et al,2020[4]	Ying Dong et al,2019[11]	Yunsong Wang et al.,2019[2]
	assessing the risk of bias (RoB) in individual studies that were included in the review?															
10	Did the review authors report on the sources of funding for the studies included in the review?	N	Y	Y	Y	Y	Y	Y	Y	N	Y	Y	Y	Y	Y	Y
11	If meta-analysis was performed, did the review authors use appropriate methods for statistical combinationof results?	**Y**	**Y**	**Y**	**Y**	**Y**	**Y**	**Y**	**Y**	**Y**	**Y**	**Y**	**Y**	**Y**	**Y**	**Y**

No	Content of the domain AMSTAR 2	Systematic Reviews and Meta-analysis from flow diagram of study selection														
		**1**	**2**	**3**	**4**	**5**	**6**	**7**	**8**	**9**	**10**	**11**	**12**	**13**	**14**	**15**
		**Alexandra C. Murphy et al.,2022[7]**	**Corinne Frere et al,2022[10]**	**Frits I. Mulder et al,2020[8]**	**Gustavo Muc oucah Sampaio Brandao**	**Haoyu Ning et al,2022[6]**	**Hiroki Ueyama et al,2020[3]**	**Irbaz Bin Riaz et al,2022[5]**	**Michela Giustozzi et al,2022[15]**	**Natale Daniele Brunetti et al,2019[13]**	**Ruchi Desai et al,2020[12]**	**Shuyi Wu et al.,2022[1]**	**V. Mai et al,2020[9]**	**Xiaojun Song et al,2020[4]**	**Ying Dong et al,2019[11]**	**Yunsong Wang et al.,2019[2]**
12	If meta-analysis was performed, did the review authors assess the potential impact of RoB in individual studies on the results of the meta-analysis or other evidence synthesis?	N	Y	Y	Y	Y	Y	Y	Y	Y	Y	Y	Y	N	N	Y
13	Did the review authors account for RoB in individual studies when interpreting/ discussing the results of the review?	**N**	**N**	**Y**	**Y**	**Y**	**Y**	**Y**	**Y**	**Y**	**Y**	**N**	**Y**	**N**	**N**	**Y**

No	Content of the domain AMSTAR 2	Systematic Reviews and Meta-analysis from flow diagram of study selection														
		**1**	**2**	**3**	**4**	**5**	**6**	**7**	**8**	**9**	**10**	**11**	**12**	**13**	**14**	**15**
		**Alexandra C. Murphy et al.,2022[7]**	**Corinne Frere et al,2022[10]**	**Frits I. Mulder et al,2020[8]**	**Gustavo Muc oucah Sampaio Brandao**	**Haoyu Ning et al,2022[6]**	**Hiroki Ueyama et al,2020[3]**	**Irbaz Bin Riaz et al,2022[5]**	**Michela Giustozzi et al,2022[15]**	**Natale Daniele Brunetti et al,2019[13]**	**Ruchi Desai et al,2020[12]**	**Shuyi Wu et al.,2022[1]**	**V. Mai et al,2020[9]**	**Xiaojun Song et al,2020[4]**	**Ying Dong et al,2019[11]**	**Yunsong Wang et al.,2019[2]**
14	Did the review authors provide a satisfactory explanation for and discussion of any heterogeneity observed in the results of the review?	N	N	Y	Y	Y	Y	Y	Y	Y	Y	Y	Y	Y	Y	Y
15	If they performed quantitative synthesis, did the review authors carry out an adequate investigation of publication bias (small study bias) and	**Y**	**Y**	**Y**	**Y**	**Y**	**Y**	**Y**	**Y**	**Y**	**Y**	**Y**	**Y**	**Y**	**Y**	**Y**

No	Content of the domain AMSTAR 2	Systematic Reviews and Meta-analysis from flow diagram of study selection														
		1	2	3	4	5	6	7	8	9	10	11	12	13	14	15
		Alexandra C. Murphy et al.,2022[7]	Corinne Frere et al,2022[10]	Frits I. Mulder et al,2020[8]	Gustavo Muc oucah Sampaio Brandao	Haoyu Ning et al,2022[6]	Hiroki Ueyama et al,2020[3]	Irbaz Bin Riaz et al,2022[5]	Michela Giustozzi et al,2022[15]	Natale Daniele Brunetti et al,2019[13]	Ruchi Desai et al,2020[12]	Shuyi Wu et al.,2022[1]	V. Mai et al,2020[9]	Xiaojun Song et al,2020[4]	Ying Dong et al,2019[11]	Yunsong Wang et al.,2019[2]
	discuss its likely impact on the results of the review?															
16	Did the review authors report any potential sources of conflict of interest, including any funding they received for conducting the review?	Y	Y	Y	Y	Y	Y	Y	Y	Y	Y	Y	Y	Y	Y	Y
Result		Low	Low	High	High	High	High	High	High	High	High	Low	High	Low	Low	High

Quality assessment of included RCTs for network meta-analysis

The Revised Cochrane Risk of Bias Tool (RoB2.0) was used for evaluating the bias of the RCTs. The degree of bias found in the individual studies were categorized into high, some concerns, or low risk of bias. The studies were judged to be at high risk of bias in at least one domain for this result or to have some concerns for multiple domains in a way that substantially lowers confidence in the result. Five RCTs[21,26,28,31,34] were classified as low risk of bias, three RCTs[20,25,30] were classified as some concerns, and four RCTs[22-24,29] were classified as high risk of bias (Supplementary I Figure 2).

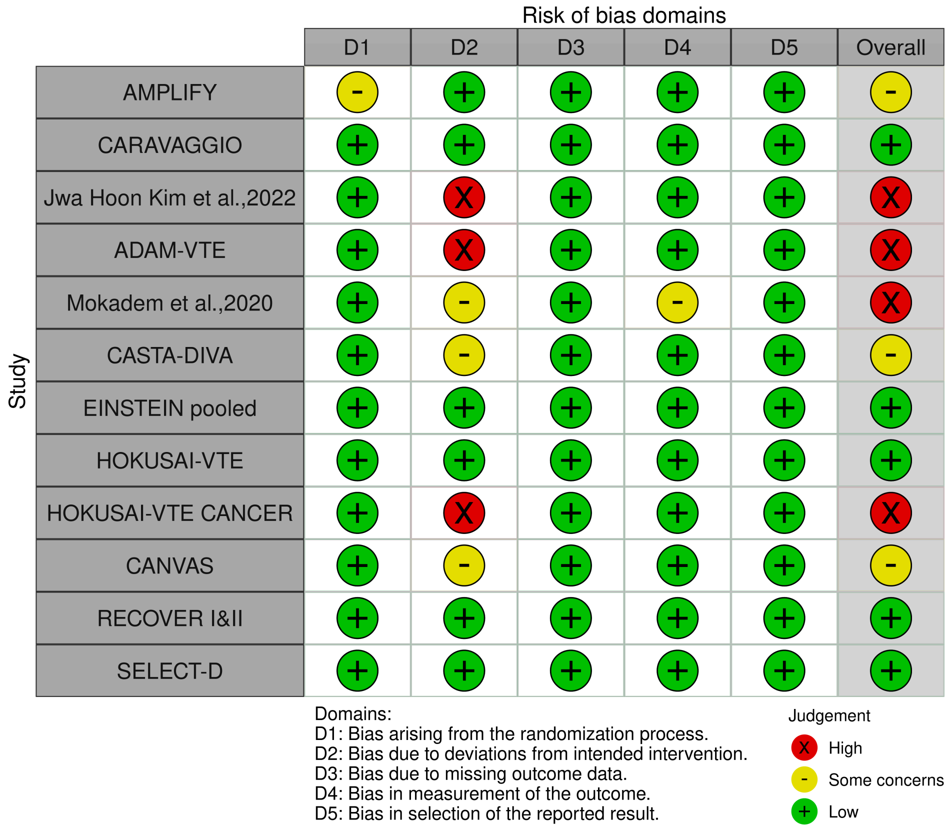

Figure S2 Risk of Bias of included RCTs

References

1. Wu S, Lv M, Chen J, et al. Direct oral anticoagulants for venous thromboembolism in cancer patients: a systematic review and network meta-analysis. *Support Care Cancer*. Dec 2022;30(12):10407-10420. doi:10.1007/s00520-022-07433-4
2. Wang Y, Lv H, Li D, et al. Efficacy and Safety of Direct Oral Anticoagulants for Secondary Prevention of Cancer-Associated Thrombosis: A Systematic Review and Meta-Analysis of Randomized Controlled Trials and Prospective Cohort Studies. *Front Pharmacol*. 2019;10:773. doi:10.3389/fphar.2019.00773
3. Ueyama H, Miyashita H, Takagi H, et al. Network meta-analysis of anticoagulation strategies for venous thromboembolism in patients with cancer. *J Thromb Thrombolysis*. Jan 2021;51(1):102-111. doi:10.1007/s11239-020-02151-2
4. Song X, Liu Z, Zeng R, et al. Treatment of venous thromboembolism in cancer patients: a systematic review and meta-analysis on the efficacy and safety of different direct oral anticoagulants (DOACs). *Ann Transl Med*. Jan 2021;9(2):162. doi:10.21037/atm-20-8156
5. Riaz IB, Fuentes HE, Naqvi SAA, et al. Direct Oral Anticoagulants Compared With Dalteparin for Treatment of Cancer-Associated Thrombosis: A Living, Interactive Systematic Review and Network Meta-analysis. *Mayo Clin Proc*. Feb 2022;97(2):308-324. doi:10.1016/j.mayocp.2020.10.041
6. Ning H, Yang N, Ding Y, et al. Efficacy and safety of direct oral anticoagulants for the treatment of cancer-associated venous thromboembolism: A systematic review and Bayesian network meta-analysis. *Med Clin (Barc)*. Mar 24 2023;160(6):245-252. doi:10.1016/j.medcli.2022.06.022
7. Murphy AC, Koshy AN, Farouque O, et al. Factor Xa Inhibition for the Treatment of Venous Thromboembolism Associated With Cancer: A Meta-Analysis of the Randomised Controlled Trials. *Heart Lung Circ*. May 2022;31(5):716-725. doi:10.1016/j.hlc.2021.10.024
8. Mulder FI, Bosch FTM, Young AM, et al. Direct oral anticoagulants for cancer-associated venous thromboembolism: a systematic review and meta-analysis. *Blood*. Sep 17 2020;136(12):1433-1441. doi:10.1182/blood.2020005819
9. Mai V, Tanguay VF, Guay CA, et al. DOAC compared to LMWH in the treatment of cancer related-venous thromboembolism: a systematic review and meta-analysis. *J Thromb Thrombolysis*. Oct 2020;50(3):661-667. doi:10.1007/s11239-020-02055-1
10. Frere C, Farge D, Schrag D, Prata PH, Connors JM. Direct oral anticoagulant versus low molecular weight heparin for the treatment of cancer-associated venous thromboembolism: 2022 updated systematic review and meta-analysis of randomized controlled trials. *J Hematol Oncol*. May 21 2022;15(1):69. doi:10.1186/s13045-022-01289-1
11. Dong Y, Wang Y, Ma RL, et al. Efficacy and safety of direct oral anticoagulants versus low-molecular-weight heparin in patients with cancer: a systematic review and meta-analysis. *J Thromb Thrombolysis*. Oct 2019;48(3):400-412. doi:10.1007/s11239-019-01871-4
12. Desai R, Koipallil GK, Thomas N, et al. Efficacy and safety of direct oral anticoagulants for secondary prevention of cancer associated thrombosis: a meta-analysis of randomized controlled trials. *Sci Rep*. Nov 3 2020;10(1):18945. doi:10.1038/s41598-020-75863-3
13. Brunetti ND, Tricarico L, Correale M, et al. Direct oral anticoagulants more effective than low-molecular-weight heparin for venous thrombo-embolism in cancer: an updated meta-analysis of randomized trials. *J Thromb Thrombolysis*. Aug 2020;50(2):305-310. doi:10.1007/s11239-019-01974-y
14. Brandão GMS, Malgor RD, Vieceli T, et al. A network meta-analysis of direct factor Xa inhibitors for the treatment of cancer-associated venous thromboembolism. *Vascular*. Feb 2022;30(1):130-145. doi:10.1177/17085381211002726

15. Giustozzi M, Agnelli G, Del Toro-Cervera J, et al. Direct Oral Anticoagulants for the Treatment of Acute Venous Thromboembolism Associated with Cancer: A Systematic Review and Meta-Analysis. *Thromb Haemost*. Jul 2020;120(7):1128-1136. doi:10.1055/s-0040-1712098
16. Awan AR, Ahmad A, Daniyal M, et al. Efficacy and Safety of Rivaroxaban Versus Enoxaparin in Prevention of Recurrence of Venous Thrombo-Embolism Events in Cancer Patients: A Meta-Analysis. *Clin Appl Thromb Hemost*. Jan-Dec 2024;30:10760296241261364. doi:10.1177/10760296241261364
17. Baloch MF, Adepoju AV, Falki V, et al. Comparative Efficacy of Oral Apixaban and Subcutaneous Low Molecular Weight Heparins in the Treatment of Cancer-Associated Thromboembolism: A Meta-Analysis. *Cureus*. Aug 2023;15(8):e43447. doi:10.7759/cureus.43447
18. Xing J, Yin X, Chen D. Rivaroxaban versus enoxaparin for the prevention of recurrent venous thromboembolism in patients with cancer: A meta-analysis. *Medicine (Baltimore)*. Aug 2018;97(31):e11384. doi:10.1097/md.0000000000011384
19. Mohamed MFH, ElShafei MN, Ahmed MB, et al. The Net Clinical Benefit of Rivaroxaban Compared to Low-Molecular-Weight Heparin in the Treatment of Cancer-Associated Thrombosis: Systematic Review and Meta-Analysis. *Clin Appl Thromb Hemost*. Jan-Dec 2021;27:1076029620940046. doi:10.1177/1076029620940046
20. Agnelli G, Buller HR, Cohen A, et al. Oral apixaban for the treatment of venous thromboembolism in cancer patients: results from the AMPLIFY trial. *J Thromb Haemost*. Dec 2015;13(12):2187-91. doi:10.1111/jth.13153
21. Agnelli G, Becattini C, Meyer G, et al. Apixaban for the Treatment of Venous Thromboembolism Associated with Cancer. *N Engl J Med*. Apr 23 2020;382(17):1599-1607. doi:10.1056/NEJMoa1915103
22. Kim JH, Yoo C, Seo S, et al. A Phase II Study to Compare the Safety and Efficacy of Direct Oral Anticoagulants versus Subcutaneous Dalteparin for Cancer-Associated Venous Thromboembolism in Patients with Advanced Upper Gastrointestinal, Hepatobiliary and Pancreatic Cancer: PRIORITY. *Cancers (Basel)*. Jan 22 2022;14(3)doi:10.3390/cancers14030559
23. McBane RD, 2nd, Wysokinski WE, Le-Rademacher JG, et al. Apixaban and dalteparin in active malignancy-associated venous thromboembolism: The ADAM VTE trial. *J Thromb Haemost*. Feb 2020;18(2):411-421. doi:10.1111/jth.14662
24. Mokadem ME, Hassan A, Algaby AZ. Efficacy and safety of apixaban in patients with active malignancy and acute deep venous thrombosis. *Vascular*. Oct 2021;29(5):745-750. doi:10.1177/1708538120971148
25. Planquette B, Bertoletti L, Charles-Nelson A, et al. Rivaroxaban vs Dalteparin in Cancer-Associated Thromboembolism: A Randomized Trial. *Chest*. Mar 2022;161(3):781-790. doi:10.1016/j.chest.2021.09.037
26. Prins MH, Lensing AW, Brighton TA, et al. Oral rivaroxaban versus enoxaparin with vitamin K antagonist for the treatment of symptomatic venous thromboembolism in patients with cancer (EINSTEIN-DVT and EINSTEIN-PE): a pooled subgroup analysis of two randomised controlled trials. *Lancet Haematol*. Oct 2014;1(1):e37-46. doi:10.1016/s2352-3026(14)70018-3
27. Prins MH, Lensing AW, Bauersachs R, et al. Oral rivaroxaban versus standard therapy for the treatment of symptomatic venous thromboembolism: a pooled analysis of the EINSTEIN-DVT and PE randomized studies. *Thromb J*. Sep 20 2013;11(1):21. doi:10.1186/1477-9560-11-21
28. Raskob GE, van Es N, Segers A, et al. Edoxaban for venous thromboembolism in patients with cancer: results from a non-inferiority subgroup analysis of the Hokusai-VTE randomised, double-blind, double-dummy trial. *Lancet Haematol*. Aug 2016;3(8):e379-87. doi:10.1016/s2352-3026(16)30057-6
29. Raskob GE, van Es N, Verhamme P, et al. Edoxaban for the Treatment of Cancer-Associated Venous Thromboembolism. *N Engl J Med*. Feb 15 2018;378(7):615-624. doi:10.1056/NEJMoa1711948

30. Schrag D, Uno H, Rosovsky R, et al. Direct Oral Anticoagulants vs Low-Molecular-Weight Heparin and Recurrent VTE in Patients With Cancer: A Randomized Clinical Trial. *Jama*. Jun 13 2023;329(22):1924-1933. doi:10.1001/jama.2023.7843
31. Schulman S, Goldhaber SZ, Kearon C, et al. Treatment with dabigatran or warfarin in patients with venous thromboembolism and cancer. *Thromb Haemost*. Jul 2015;114(1):150-7. doi:10.1160/th14-11-0977
32. Schulman S, Kakkar AK, Goldhaber SZ, et al. Treatment of acute venous thromboembolism with dabigatran or warfarin and pooled analysis. *Circulation*. Feb 18 2014;129(7):764-72. doi:10.1161/circulationaha.113.004450
33. Schulman S, Kearon C, Kakkar AK, et al. Dabigatran versus warfarin in the treatment of acute venous thromboembolism. *N Engl J Med*. Dec 10 2009;361(24):2342-52. doi:10.1056/NEJMoa0906598
34. Young AM, Marshall A, Thirlwall J, et al. Comparison of an Oral Factor Xa Inhibitor With Low Molecular Weight Heparin in Patients With Cancer With Venous Thromboembolism: Results of a Randomized Trial (SELECT-D). *J Clin Oncol*. Jul 10 2018;36(20):2017-2023. doi:10.1200/jco.2018.78.8034

Supplementary II

Table S4 Diagnosis Codes Used to Identify the VTE and Bleeding Events

Healthstate (Markov model)	Diagnostic	ICD-10-CM Code
	**VTE**	
Deep vein thrombosis (DVT)	Deep vein thrombosis	I80, I82
Pulmonary embolism (PE)	Pulmonary embolism	I26, I27
	**Major bleeding**	
Intracranial hemorrhage (ICH)	Intracranial hemorrhage	I60, I61, I62
	Non intracranial hemorrhage	
Non-ICH Major Bleed (MB)	Gastrointestinal bleeding	K92x, K250, K252, K262, K270, K272, K280, K290, I850, K253, K254, K256, K259, K260, K264, K266, K267, 269, K274, K279, K289, K625, K226
	Another critical site bleeding	D62, J942, H113, H356, H431, N02, R04, R31, R58, S06, G95, T780, T810
	**Clinically relevant non-major bleeding (CRNMB)**	H313, N930, N9300, N9301, N938, N939

Abbreviations: ICD-10-CM, International Classification of Diseases-10 Clinical Modification; VTE, venous thromboembolism; PE, pulmonary embolism; DVT, deep vein thrombosis; GI, gastrointestinal; ICH, intracranial haemorrhage; CRNMB bleeding Clinically relevant non-major bleeding

Supplementary III

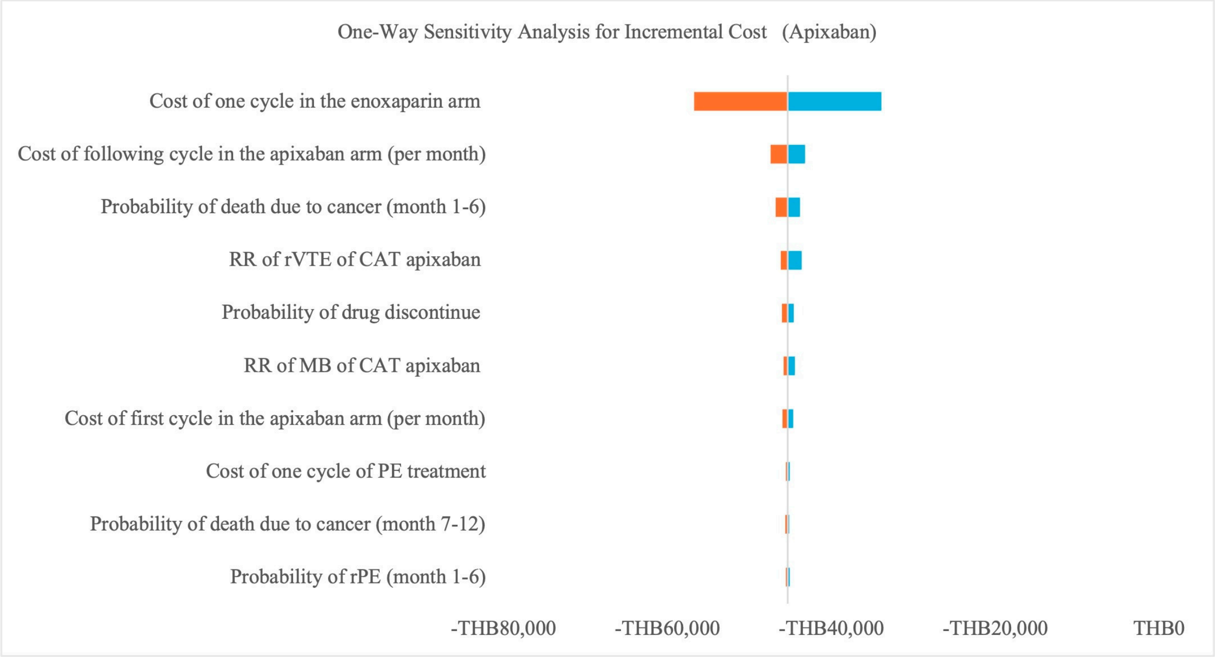

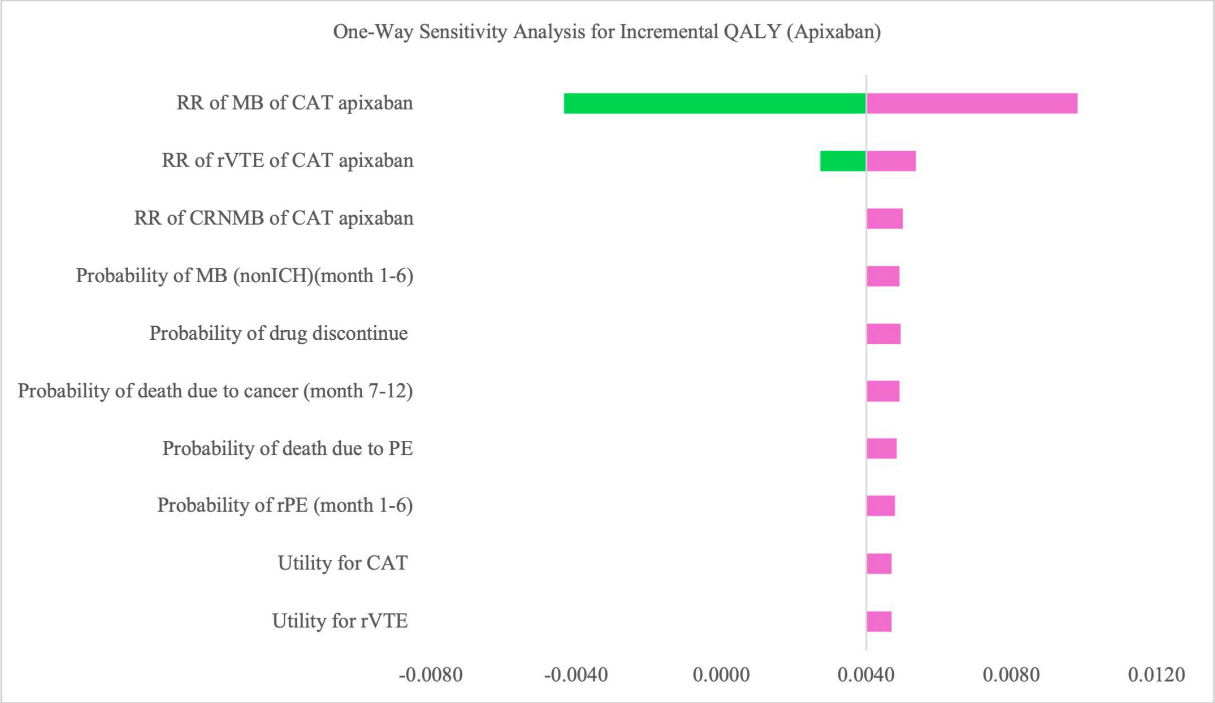

Figure S3 Tornado diagram for deterministic sensitivity analyses for incremental cost and QALY of apixaban compare to LMWH

Abbreviations: CAT, cancer-associated thrombosis; rVTE, recurrent venous thromboembolism; PE, pulmonary embolism; rPE, recurrent pulmonary embolism; DVT, deep venous thrombosis; CRNMB, clinically relevant non-major bleeding; MB, major bleeding; ICH, intracranial hemorrhage; RR, risk ratio; QALY, quality-adjusted life year.

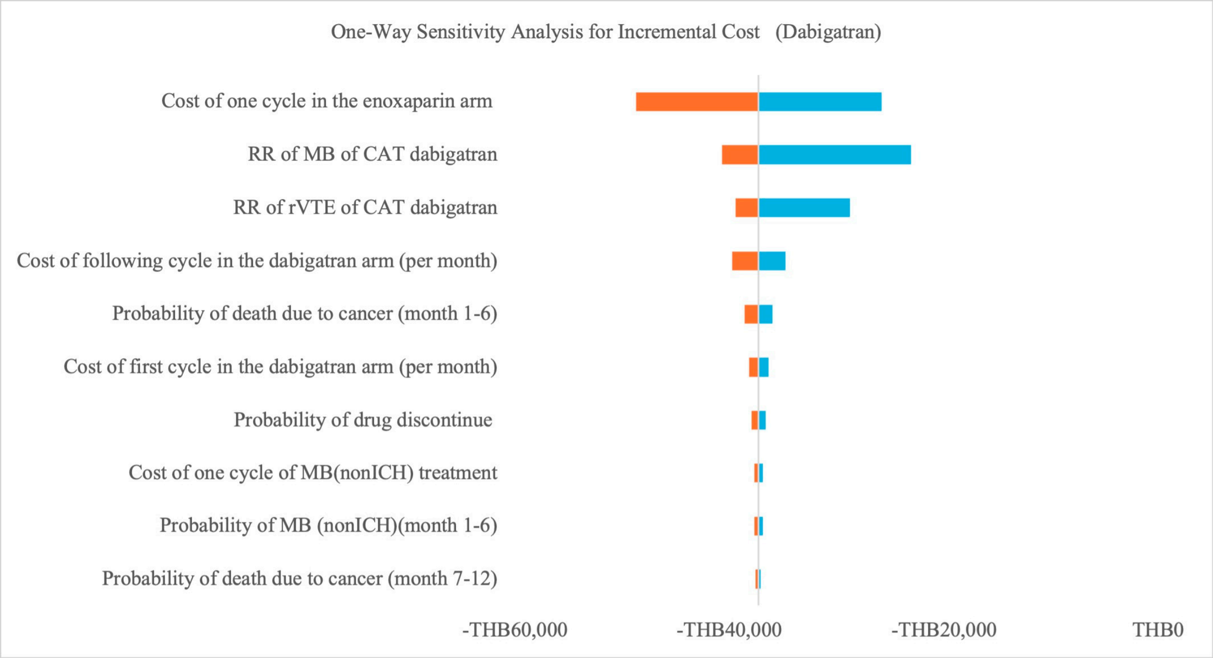

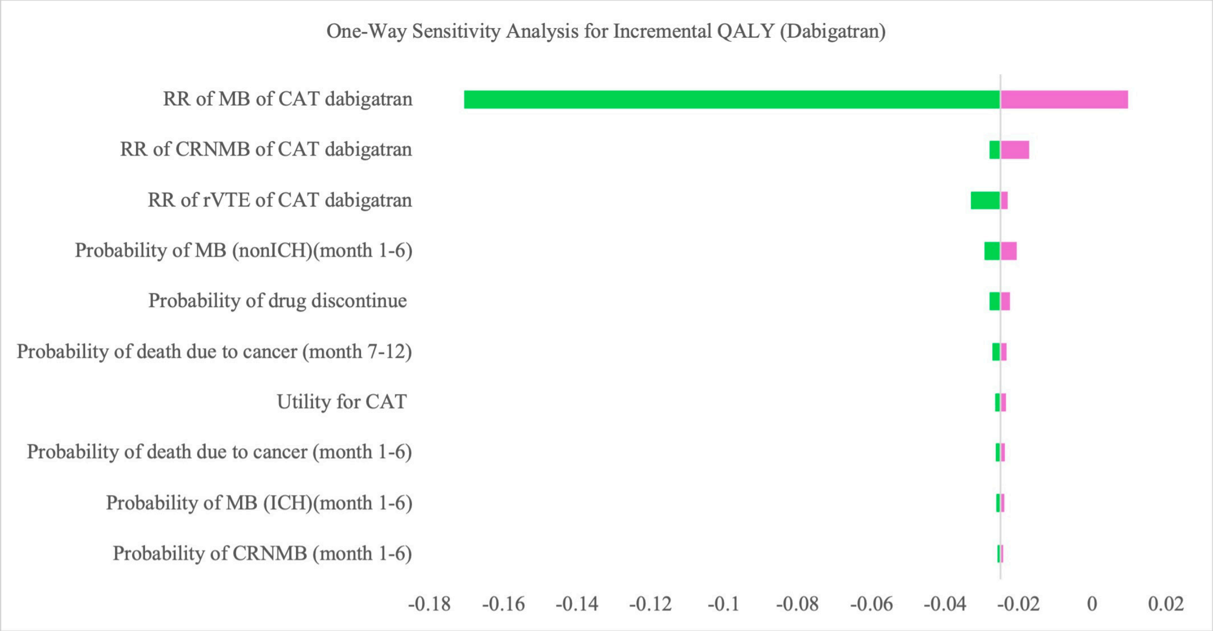

Figure S4 Tornado diagram for deterministic sensitivity analyses for incremental cost and QALY of dabigatran compared to LMWH

Abbreviations: CAT, cancer-associated thrombosis; rVTE, recurrent venous thromboembolism; PE, pulmonary embolism; rPE, recurrent pulmonary embolism; DVT, deep venous thrombosis; CRNMB, clinically relevant non-major bleeding; MB, major bleeding; ICH, intracranial hemorrhage; RR, risk ratio; QALY, quality-adjusted life year.

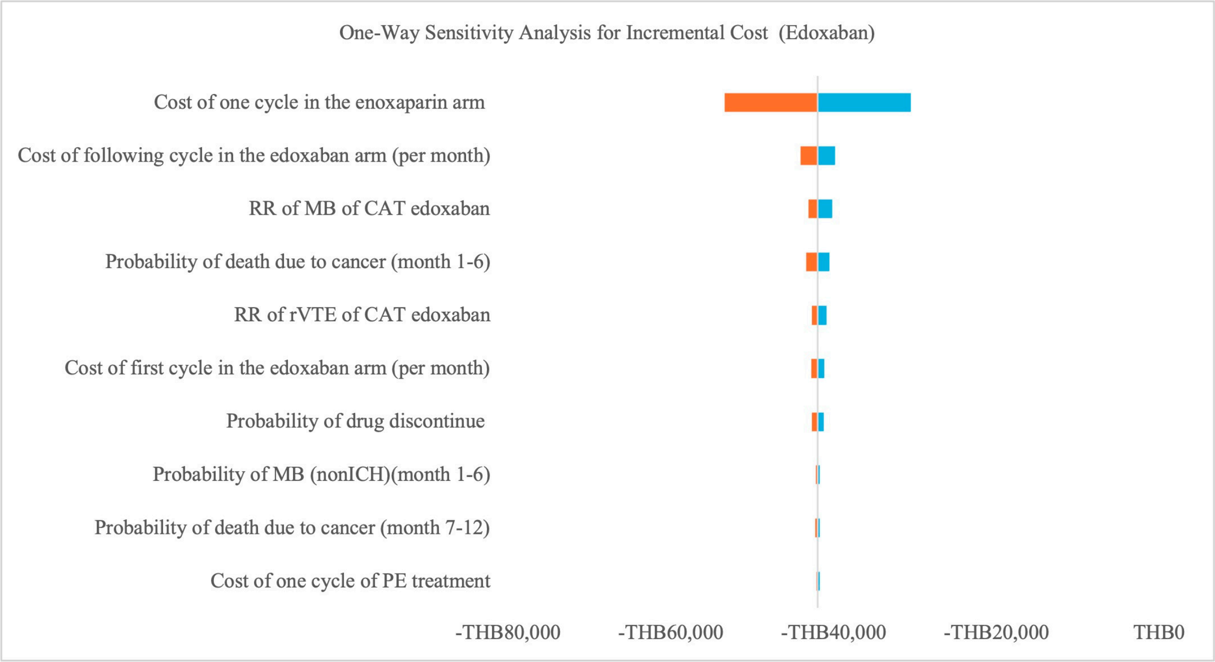

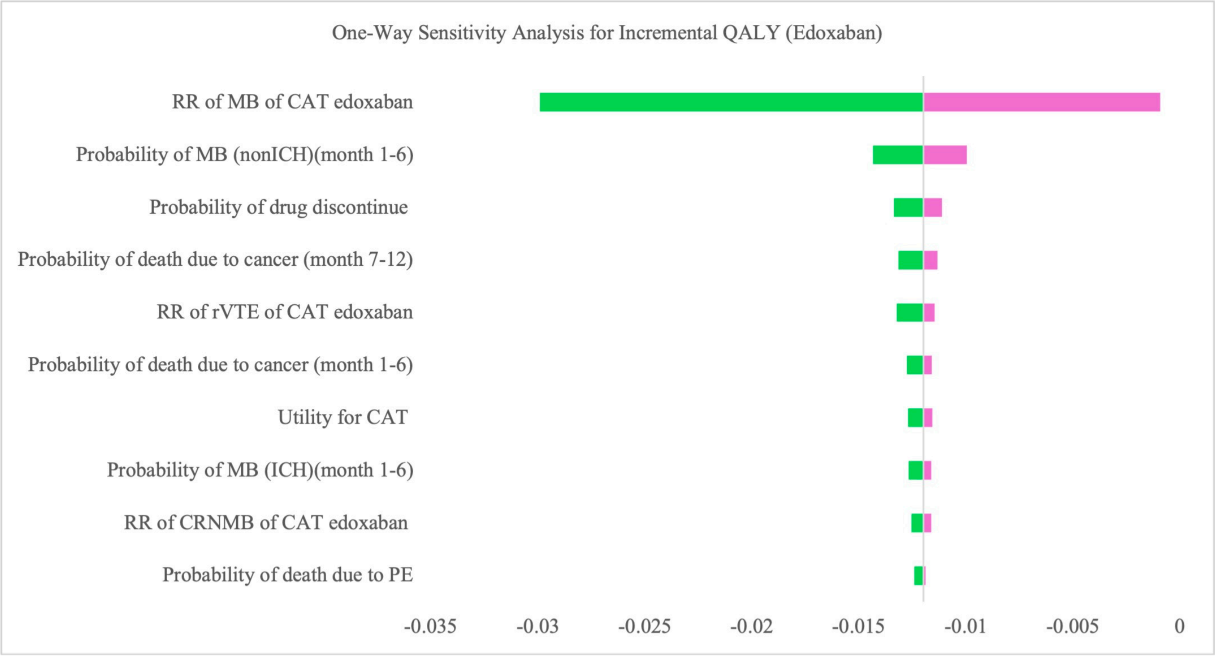

Figure S5 Tornado diagram for deterministic sensitivity analyses for incremental cost and QALY of edoxaban compared to LMWH

Abbreviations: CAT, cancer-associated thrombosis; rVTE, recurrent venous thromboembolism; PE, pulmonary embolism; rPE, recurrent pulmonary embolism; DVT, deep venous thrombosis; CRNMB,

clinically relevant non-major bleeding; MB, major bleeding; ICH, intracranial hemorrhage; RR, risk ratio; QALY, quality-adjusted life year.

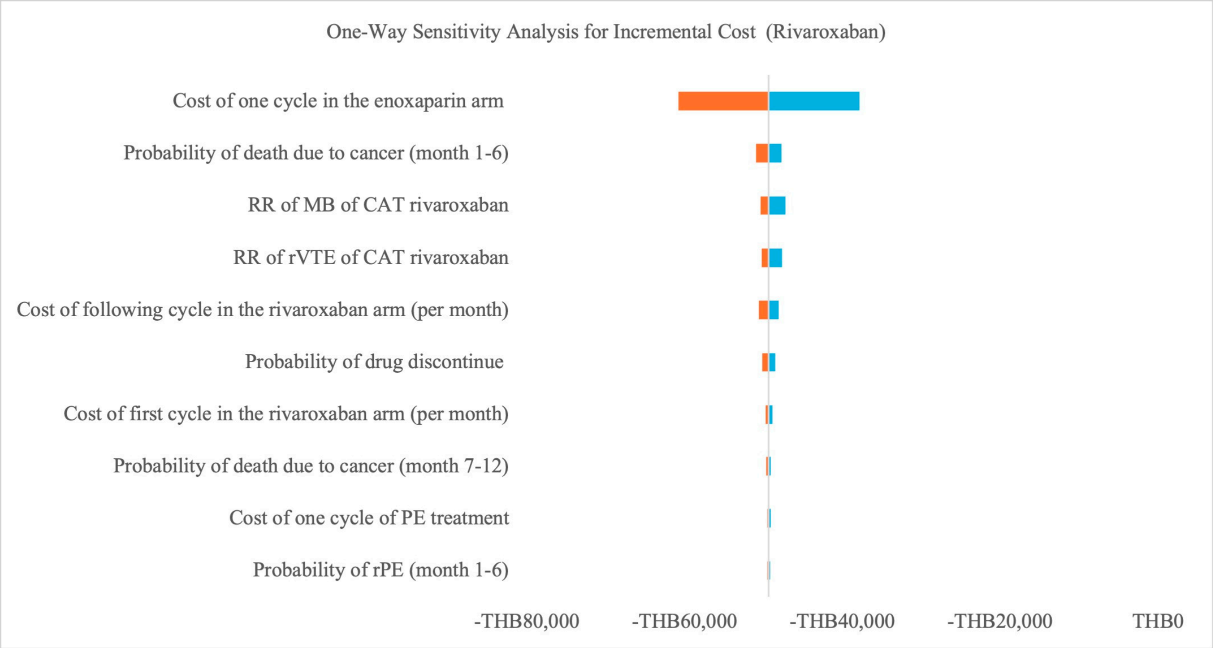

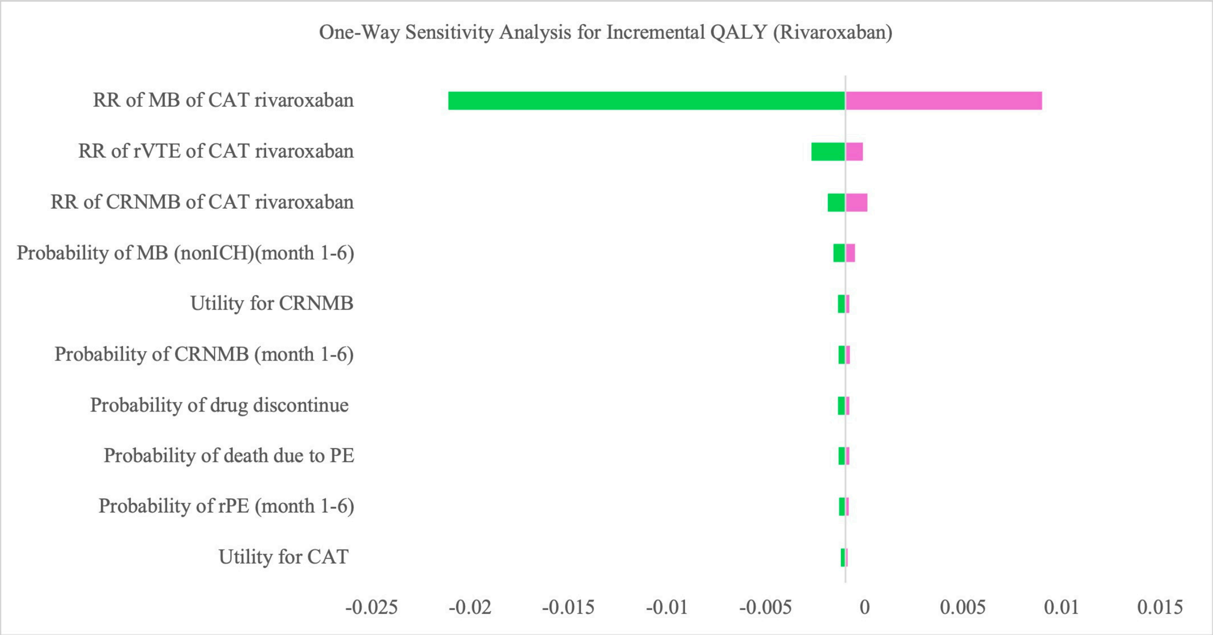

Figure S6 Tornado diagram for deterministic sensitivity analyses for incremental cost and QALY of rivaroxaban compared to LMWH

Abbreviations: CAT, cancer-associated thrombosis; rVTE, recurrent venous thromboembolism; PE, pulmonary embolism; rPE, recurrent pulmonary embolism; DVT, deep venous thrombosis; CRNMB,

clinically relevant non-major bleeding; MB, major bleeding; ICH, intracranial hemorrhage; RR, risk ratio; QALY, quality-adjusted life year.

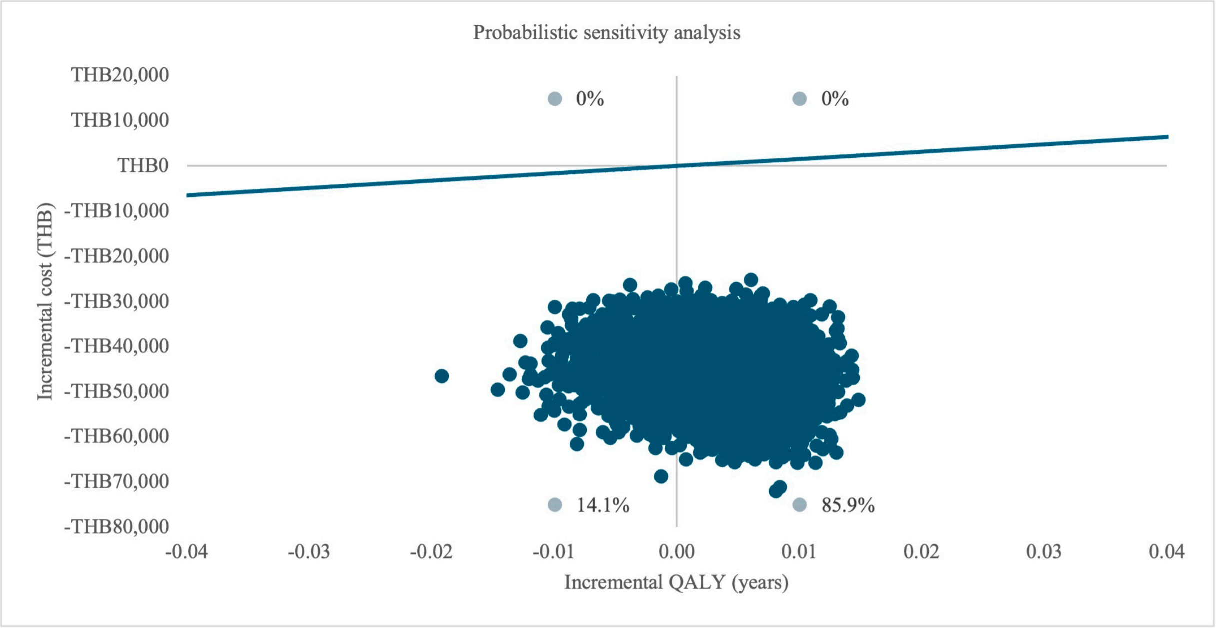

Figure S7 Probabilistic cost effectiveness plane results of apixaban compare to LMWH

Abbreviations: THB, Thai baht; QALY, quality-adjusted life year.

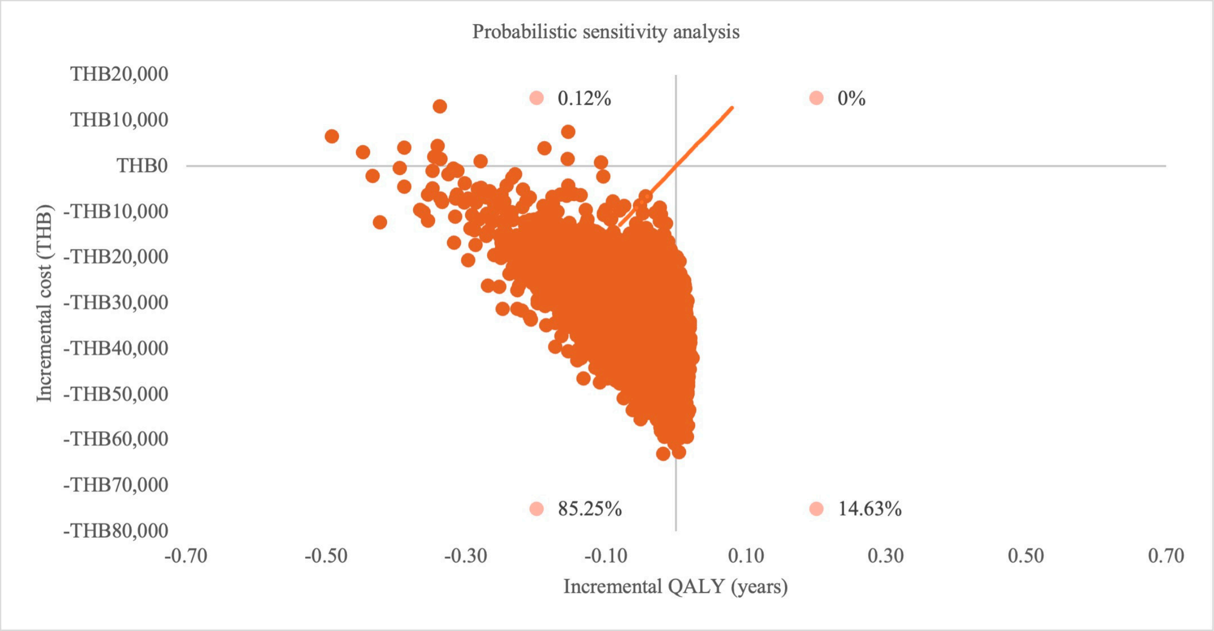

Figure S8 Probabilistic cost effectiveness plane results of dabigatran compared to LMWH

Abbreviations: THB, Thai baht; QALY, quality-adjusted life year.

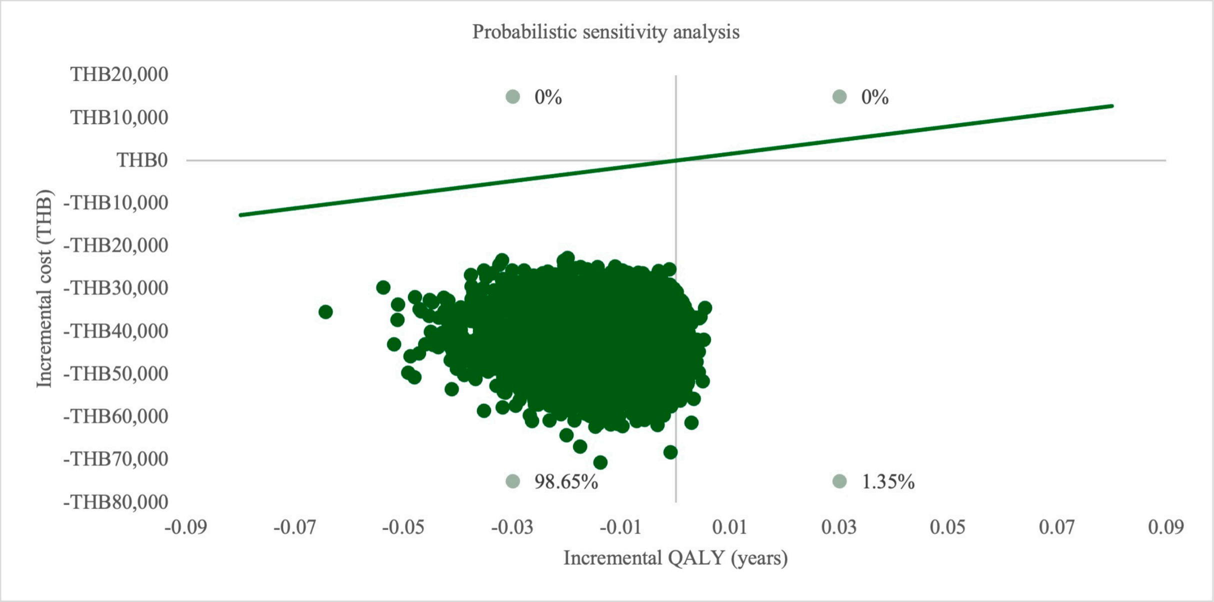

Figure S9 Probabilistic cost effectiveness plane results of edoxaban compared to LMWH

Abbreviations: THB, Thai baht; QALY, quality-adjusted life year.

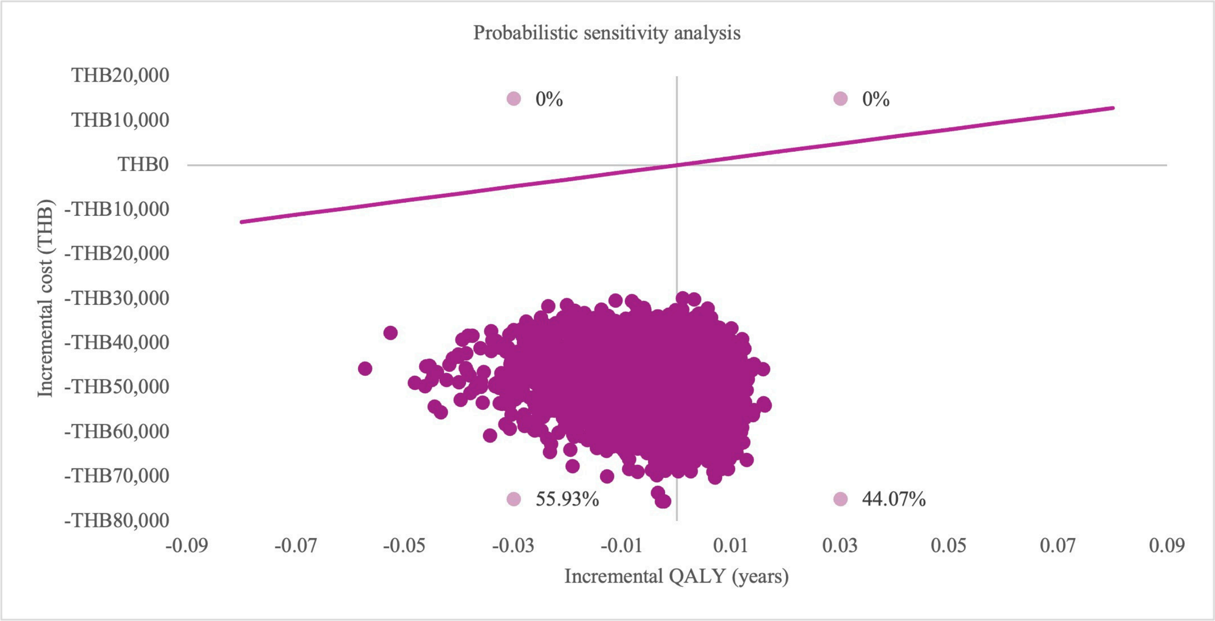

Figure S10 Probabilistic cost effectiveness plane results of rivaroxaban compared to LMWH

Abbreviations: THB, Thai baht; QALY, quality-adjusted life year.
